# D-mannose suppresses macrophage IL-1β production

**DOI:** 10.1038/s41467-020-20164-6

**Published:** 2020-12-11

**Authors:** Simone Torretta, Alessandra Scagliola, Luisa Ricci, Francesco Mainini, Sabrina Di Marco, Ivan Cuccovillo, Anna Kajaste-Rudnitski, David Sumpton, Kevin M. Ryan, Simone Cardaci

**Affiliations:** 1 Cancer Metabolism Unit, Division of Genetics and Cell Biology, IRCCS San Raffaele Scientific Institute, 20132 Milan, Italy; 2 San Raffaele Telethon Institute for Gene Therapy, IRCCS San Raffaele Scientific Institute, 20132 Milan, Italy; 3 CRUK Beatson Institute, Glasgow, UK

**Keywords:** Metabolomics, Metabolic pathways, Inflammation, Monocytes and macrophages

## Abstract

D-mannose is a monosaccharide approximately a hundred times less abundant than glucose in human blood. Previous studies demonstrated that supraphysiological levels of D-mannose inhibit tumour growth and stimulate regulatory T cell differentiation. It is not known whether D-mannose metabolism affects the function of non-proliferative cells, such as inflammatory macrophages. Here, we show that D-mannose suppresses LPS-induced macrophage activation by impairing IL-1β production. In vivo, mannose administration improves survival in a mouse model of LPS-induced endotoxemia as well as decreases progression in a mouse model of DSS-induced colitis. Phosphomannose isomerase controls response of LPS-activated macrophages to D-mannose, which impairs glucose metabolism by raising intracellular mannose-6-phosphate levels. Such alterations result in the suppression of succinate-mediated HIF-1α activation, imposing a consequent reduction of LPS-induced *Il1b* expression. Disclosing an unrecognized metabolic hijack of macrophage activation, our study points towards safe D-mannose utilization as an effective intervention against inflammatory conditions.

## Abstract (web summary)

Mannose is present at trace levels in blood and regulates cancer growth. Here the authors show that supraphysiological levels of mannose can also regulate macrophages, limiting their production of IL-1β and increasing resistance of mice to LPS-induced endotoxemia and DSS-induced colitis.

## Introduction

Macrophages are frontline cells of innate immunity, playing critical roles in the early promotion as well as resolution of inflammation. Such functional plasticity depends on specific changes in their metabolic programs. An increase in glycolysis is required to produce inflammatory cytokines, such as interleukin (IL)-1β, in response to the Toll-like receptor 4-ligand lipopolysaccharide (LPS)^1,2^. Pharmacological inhibition of glycolysis induced, for instance, by the hexokinase inhibitor 2-deoxyglucose (2-DG), results in a decrease of macrophage pro-inflammatory cytokine production^3,4^. Similarly, the immunosuppressive cytokine IL-10 inhibits effector functions of classically activated macrophages by decreasing glycolysis^5^. Consistent with the impact on inflammatory response, glycolysis inhibition offers protection in mouse models of experimental endotoxemia and a large variety of immunopathologies^3,4,6–8^. The increase of glycolytic flux is necessary to fulfill bioenergetic needs of pro-inflammatory macrophages. Indeed, whereas resting and alternatively activated macrophages predominantly generate ATP via oxidative phosphorylation, LPS decreases mitochondrial oxygen consumption, repurposing mitochondria from ATP synthesis to reactive oxygen species (ROS) production, to promote a pro-inflammatory state^9^. Beyond a rapid and robust ATP generation, a high glycolytic rate also provides biosynthetic intermediates to support macrophage effector functions. For instance, glucose fuels the pentose phosphate pathway, necessary for maintenance of redox homeostasis during inflammatory response^2^, as well as sustains levels of the non-essential amino acid serine, whose de novo synthesis supports LPS-induced IL-1β production^10,11^. Importantly, glycolysis promotes IL-1β production also by sustaining succinate levels in pro-inflammatory macrophages^3,9^. Succinate is a prototypical pro-inflammatory tricarboxylic acid (TCA) cycle intermediate accumulating in macrophages in response to LPS^3,12–14^. Such metabolite is also found increased in fluids and tissues of individuals affected by a large variety of immunopathologies, including chronic forms of intestinal inflammation, and has instrumental roles in their pathogenesis^14–16^. Mechanistically, succinate drives inflammatory signaling by inducing stabilization of the hypoxia-inducible factor 1α (HIF-1α), which, in turn, leads to up-regulation of *IL1b* gene transcription^3,9^. Beyond acting as a direct inhibitor of prolyl hydroxylases, which prime HIF-1α for proteasomal degradation, succinate may also contribute to stabilize HIF-1α by sustaining production of mitochondrial ROS through a reverse flow of electron transfer across respiratory complexes, in pro-inflammatory macrophages^9^. In line with its role in macrophage activation, myeloid deletion of *Hif1a* gene results in a decreased production of pro-inflammatory mediators and extended survival in endotoxemic mice^4,17^. Overall, this body of knowledge indicates that interventions aimed at tuning glucose utilization as well as accumulation and bioactivity of succinate in inflammatory cells might offer improved clinical benefit in immunopathology.

d-Mannose (hereafter referred to as mannose) is a natural C-2 epimer of glucose. It is transported into mammalian cells via plasma membrane facilitated diffusion glucose transporters (GLUT) and, within the cell, it is phosphorylated by hexokinase to produce mannose-6-phosphate, which may undergo two major metabolic fates. A minor fraction is isomerized to mannose-1-phosphate by phosphomannomutase, to be directed into glycosylation pathways. The large majority (~95%), instead, is converted to fructose-6-phophate by phosphomannose isomerase (MPI), to be catabolized into glycolysis. However, as physiological mannose concentration in human blood (~50 μM) is one-hundredth of that of glucose, it does not contribute significantly to cell bioenergetics^18^.

Mannose supplementation, at safe supraphysiological doses, was shown to ameliorate some human disease states. It is therapeutically effective as a non-antibiotic treatment for recurrent urinary tract infections in humans, by preventing adhesion of enteric bacteria to uroepithelial cells^18,19^. Mannose was also demonstrated to affect T cell function by promoting generation of regulatory T cells (T_regs_) from naive CD4^+^ T lymphocytes and, in such a way, suppressing T cell-mediated experimental immunopathologies in mice^20^. More recently, we reported that oral administration of mannose in mice is effective in decreasing in vivo growth of multiple tumor types and in potentiating the effects of cytotoxic chemotherapies^21^. In particular, MPI activity dictates sensitivity of tumors to the anti-proliferative effects of such sugar. Cancer cells with low MPI levels are unable to catabolize efficiently mannose-6-phosphate, which accumulates intracellularly and impairs glucose utilization^21^. Currently, it is not known whether mannose metabolism affects function of glucose-addicted non-proliferating cells, such as inflammatory macrophages.

Here, we demonstrate that mannose opposes LPS-induced macrophage activation by impairing *Il1b* gene expression. Such effect results from inhibition of glucose metabolism and suppression of succinate-mediated HIF-1α activation. Moreover, we identify MPI as the metabolic gatekeeper dictating response of macrophages to the anti-inflammatory effects of mannose and provide evidence for the utilization of such sugar to ameliorate progression of ulcerative colitis in mice, by exploiting its capability to impair pro-inflammatory metabolic activation of myeloid cells.

## Results

### Mannose opposes macrophage activation by impairing IL-1β production

**Fig. 1:**
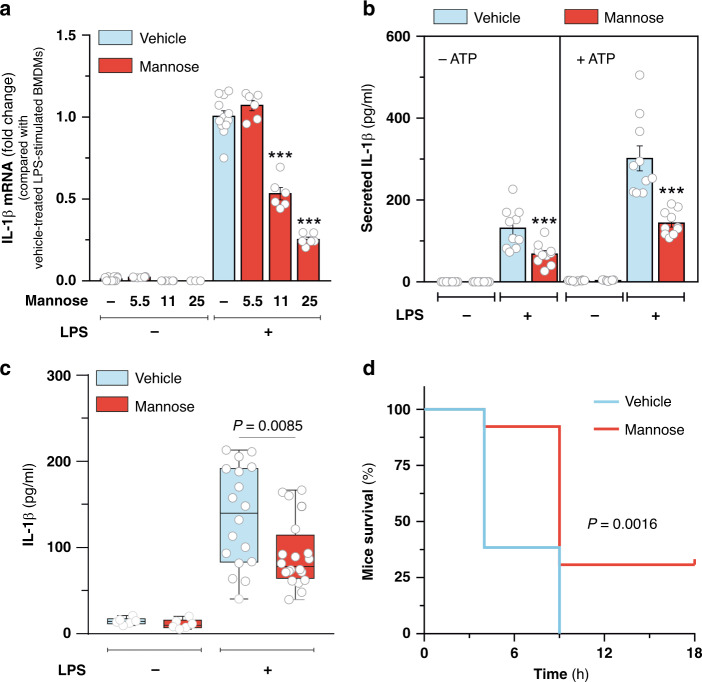
Mannose impairs LPS-induced IL-1β production. **a** qPCR analysis of IL-1β mRNA levels in resting and LPS-activated BMDMs cultured for 24 h in the presence or absence of the indicated concentrations of mannose in RPMI medium containing 11 mM glucose. Data are presented as mean ± s.e.m. of at least *n* = 6 wells pooled from two independent experiments performed in technical triplicate. In this and all other figures, “wells” represent technical replicate samples setup and assessed under identical conditions within a single experiment. ***P < 0.001 (two-tailed Student’s *t*-test). **b** ELISA-mediated determination of IL-1β secreted in media of BMDMs treated as in **a**. When indicated (+ATP), BMDMs were incubated with 2.5 mM ATP for additional 40 min for inflammasome activation. Data are presented as mean ± s.e.m. of *n* = 10 wells pooled from two independent experiments. ***P < 0.001 (two-tailed Student’s *t*-test). **c**, **d** Endotoxin-induced model of sepsis. Seven- to 9-week-aged male Balb/c mice were stimulated with a single intraperitoneal (i.p) injection of 20 mg/kg O55:B5 LPS and treated with either 2 g/kg mannose or saline control (vehicle) solution given hourly by i.p. injections for up to six times. In **c** mice were sacrificed after 3 h and IL-1β concentration in serum determined by ELISA. Data are presented as box plot of *n* = 18 (vehicle-treated LPS-stimulated), *n* = 20 (mannose-treated LPS-stimulated) and *n* = 6 (LPS untreated) mice. Boxes extend from the 25th to 75th percentiles, bold black line indicates median, and the whiskers range from the minimum to maximum value. In **d**, survival of *n* = 13 LPS-stimulated mice per group, treated as in **c**, was monitored up to 18 h. Indicated *P* value in **c** and in **d** were calculated by two-tailed Student’s *t*-test and log-rank (Mantel–Cox) test, respectively. Source data are provided as a Source Data file.

Glucose metabolism drives pro-inflammatory cytokine IL-1β production in macrophages. To determine whether mannose also has a role in macrophage activation, we treated mouse bone marrow-derived macrophages (BMDMs) with LPS for 24 h in culture media supplemented with different concentrations of mannose. Such sugar decreased LPS-induced *Il1b* gene expression dose dependently (Fig. 1a and Supplementary Fig. 1A). In particular, a minimal dose of mannose equimolar to that of glucose present in culture media was found to be effective in dampening gene expression (Fig. 1a and Supplementary Fig. 1A) and production of IL-1β (Fig. 1b) upon LPS stimulation, preserving BMDMs viability (Supplementary Fig. 1B). It is noteworthy that mannose halved LPS-induced secretion of IL-1β independently on exposure of BMDMs to an inflammasome assembly stimulus (ATP) (Fig. 1b). In line with such result, no major direct impact of mannose on caspase-1 activity was detected upon ATP-dependent inflammasome activation in LPS-stimulated BMDMs (Supplementary Fig. 1C), indicating that mannose opposes IL-1β production mainly by altering *Il1b* gene expression, rather than affecting pro-IL1β processing. Interestingly, LPS-induced *Il1b* gene expression was not reduced when mannose was replaced with equimolar concentrations of another monosaccharide, such as glucose or fructose, or mannitol, a mannose derivative not metabolized in animals (Supplementary Fig. 1D). Comparatively to *Il1b*, mannose induced a small decrease in *Tnf* and *Il6* genes expression (Supplementary Fig. 1E) and did not elicit major changes in anti-inflammatory cytokines transcription (Supplementary Fig. 1F) in LPS-stimulated BMDMs. Similar effects on pro-inflammatory cytokine profile were also observed in BMDMs generated from a different mouse strain (Balb/c) (Supplementary Fig. 1G), RAW 264.7 murine macrophage cell line (Supplementary Fig. 1H) as well as human macrophages derived from peripheral blood monocytes (Supplementary Fig. 1I, J). Collectively, these data indicate that mannose opposes classical macrophage activation by limiting, preferentially, optimal IL-1β production.

Prompted by these data, we investigated whether mannose had an in inhibitory effect on the systemic pro-inflammatory response to LPS in vivo. Administration of 2 g/kg mannose in mice, given hourly via intraperitoneal injections, resulted in a blood mannose concentration of ~6.8–9.5 mM (Supplementary Fig. 2A) and was effective in decreasing serum levels of IL-1β in mice challenged with a single lethal dose of LPS (Fig. 1c). Importantly, mannose treatment did not change weight of unstimulated mice (Supplementary Fig. 2B), nor did it obviously affect their normal behavior and health. However, it significantly prolonged survival rate of LPS-treated mice (Fig. 1d). Overall, these data indicate that well tolerated mannose administration regimen offers protection from experimental lethal endotoxemia, associated with decreased systemic IL-1β release.

### Mannose impairs glucose utilization and succinate-mediated HIF-1α activation

Next, we investigated the mechanisms by which mannose impairs LPS-induced IL-1β production. The mannose receptor (MRC1) is a C-type lectin expressed in phagocytes capable to bind microbial glycoligands containing exposed mannose or other sugar residues^22^. As MRC1 might play important roles in control of inflammation, we measured the extent to which it contributes to mannose-mediated suppression of IL1β production. Silencing MRC1 expression, achieved by transducing BMDMs with two independent short hairpin RNAs (shRNAs) (Supplementary Fig. 3A, B), did not abolish the relative ability of mannose treatment to decrease IL1β production in LPS-stimulated macrophages (Supplementary Fig. 3C), suggesting that mannose opposes IL-1β production primarily via an MRC1-independent manner.

**Fig. 2:**
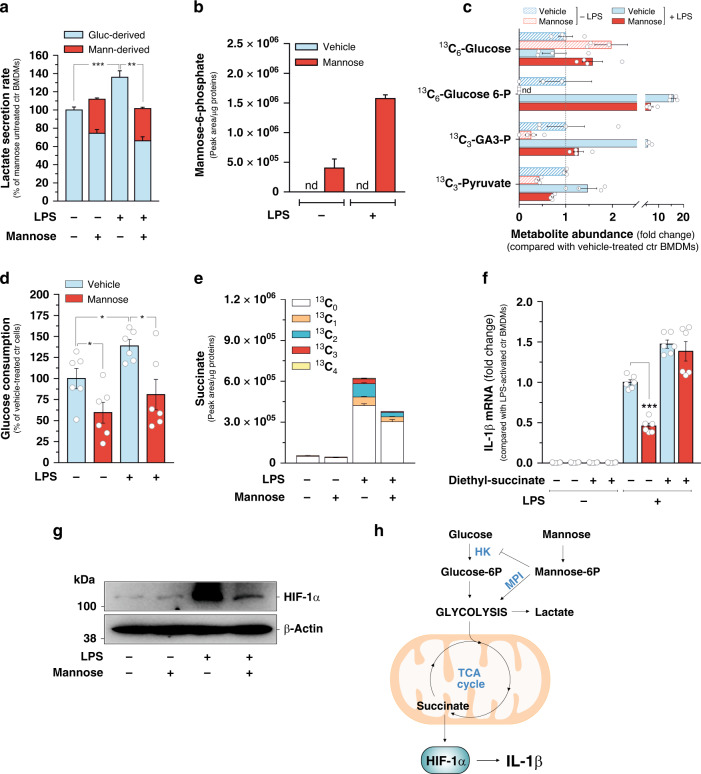
Mannose impairs glucose utilization and succinate-mediated HIF-1α activation. **a** Lactate secretion rate measured in resting and LPS-activated BMDMs cultured for 24 h in RPMI medium containing 11 mM glucose in the presence/absence of 11 mM U-^13^C_6_-Mannose. ^12^C-lactate and ^13^C_3_-lactate were used were used as identifiers of glucose (Gluc-derived) and mannose-derived (Mann-derived) lactate, respectively. Data are presented as mean ± s.e.m. of *n* = 6 wells pooled from two independent experiments. **b** Intracellular levels of mannose-6-phosphate (^13^C_6_-mannose-6-phosphate) measured in BMDMs cultured for 24 h in RPMI medium containing 11 mM glucose in the presence/absence of 11 mM U-^13^C_6_-mannose. Data are presented as mean ± s.e.m. of *n* = 4 wells from two independent experiments; nd not detectable. **c** Intracellular levels of the indicated metabolites in BMDMs cultured for 24 h in RPMI medium containing 11 mM U-^13^C_6_-glucose in the presence/absence of 11 mM mannose. Data are presented as mean ± s.e.m. of *n* = 4 wells from two independent experiments. ^13^C_3_-Glucose-6-P ^13^C_3_-Glucose-6-phosphate, ^13^C_3_-GA3-P ^13^C_3_-glyceraldehyde-3-phosphate, nd not detectable. **d** Glucose consumption rate in BMDMs cultured for 24 h as in **b**. Data are presented as mean ± s.e.m. of *n* = 6 wells pooled from two independent experiments. **e** Intracellular succinate levels in BMDMs cultured for 24 h RPMI medium containing in 11 mM U-^13^C_6_-Glucose in the presence/absence of 11 mM mannose. The abundance of all succinate isotopologues is reported. Data are presented as mean ± s.e.m. of *n* = 4 wells from two independent experiments. **f** Effect of 5 mM diethyl-succinate treatment in BMDMs on IL-1β mRNA levels measured in BMDMs cultured for 24 h in medium containing in 11 mM glucose in the presence/absence of 11 mM mannose. Data are presented as mean ± s.e.m. of *n* = 6 wells pooled from two independent experiments performed in technical triplicate. **g** Representative image of HIF-1α of two independent experiments in BMDMs cultured for 24 h in RPMI medium containing 11 mM glucose in the presence/absence of 11 mM mannose. β-Actin was used as a loading control. **h** Representation of glycolysis, TCA cycle, reactions metabolizing mannose into glycolysis and regulation of *Il1b* expression by succinate-mediated HIF-1α activation. HK hexokinase, MPI phosphomannose isomerase. In **a**, **d**, **f** *P < 0.05, **P < 0.01 and ***P < 0.001 (two-tailed Student’s *t*-test). Source data are provided as a Source Data file.

We have recently demonstrated that mannose decreases proliferative capacity of cancer cells by inhibiting glycolysis^21^. Interestingly, mannose was found to be more effective in inhibiting *Il1b* expression in BMDMs cultured in a medium containing low glucose (5.5 mM) compared with a medium with standard (higher) glucose concentration (11 mM) (Fig. 1a and Supplementary Fig. 1A), suggesting that it might control *Il1b* expression in LPS-activated macrophages by affecting glucose metabolism. Therefore, prompted by such results, we determined directly whether mannose had impact on macrophage glucose metabolism by stimulating BMDMs with LPS for 24 h in the presence or absence of uniformly labeled U-^13^C_6_-mannose in the culture medium, in order to distinguish metabolites containing mannose carbons from those derived from glucose, by molecular mass. Liquid chromatography-mass spectrometry (LC-MS) analysis indicated that mannose treatment opposes the increase of glycolytic rate induced by pro-inflammatory stimulation in macrophages, as shown by the decreased lactate secretion in LPS-activated BMDMs incubated with mannose, with respect to mannose-untreated counterparts (Fig. 2a). Moreover, compared with glucose, mannose is less efficiently metabolized through glycolysis in macrophages, as evidenced by the lower fractional secretion rate of lactate pool derived from mannose (^13^C_3_-lactate), with respect to that produced from glucose (^12^C-lactate), in mannose-treated BMDMs (Fig. 2a), and accumulates intracellularly as mannose-6-phosphate (Fig. 2b), whose levels are further increased in inflammatory macrophages, reasonably as a result of higher mannose uptake rate detected in in response to LPS (Supplementary Fig. 4A). Mannose-6-phosphate is known to act as an inhibitor of some apical glycolytic enzymes, such as hexokinase and phosphoglucose isomerase^23^. Therefore, to elucidate the impact of mannose on glucose metabolism in macrophages, we traced the intracellular fate of glucose carbons in BMDMs cultured in a medium containing uniformly labeled U-^13^C_6_-glucose. LC-MS-mediated deconvolution of glycolytic intermediates revealed that mannose treatment mainly hampers glucose phosphorylation rather than its uptake in macrophages, as indicated by the increase in intracellular ^13^C_6_-glucose abundance and concomitant decrease in the levels of ^13^C_6_-glucose-6-phosphate in mannose-treated BMDMs, compared with mannose-untreated counterparts (Fig. 2d). As a result of such alteration, mannose supplementation significantly decreased the intracellular abundance of representative glucose-derived glycolytic metabolites downstream glucose-6-phosphate (Fig. 2d), and resulted in a drop of glucose consumption rate (Fig. 2e). In line with the reduction of the overall glycolytic flux, a significant attenuation of ATP levels was measured in LPS-activated BMDMs incubated with mannose, with respect to mannose-untreated macrophages (Supplementary Fig. 4B). In line with such metabolic changes, incubation of BMDMs with the downstream glycolysis intermediate pyruvate, provided in the form of its cell-permeable analog methyl-pyruvate, repressed substantially the capability of mannose to oppose IL-1β production upon LPS exposure (Supplementary Fig. 4C), indicating that the impairment of glycolytic route is instrumental for the effects of mannose treatment on macrophage activation.

Glucose is a key carbon source for the TCA cycle, which acts as a metabolic platform for inflammatory signaling in macrophages^24^. Consistent with the decreased utilization of glucose in glycolysis, mannose treatment reduced also the entry of glucose-derived carbons into the TCA cycle, as demonstrated by the considerable suppression of ^13^C labeling pattern of several TCA cycle metabolites both in mannose-treated resting and LPS-stimulated BMDMs cultured in U-^13^C_6_-glucose-containing medium, with respect to mannose-untreated counterparts (Fig. 2e and Supplementary Fig. 4D, E). Suppression of glucose catabolism is known to decrease succinate abundance in inflammatory macrophages^3^. Consistent with this, the diminished glucose-dependent TCA cycle anaplerosis was associated with a substantial attenuation of succinate levels accumulating in BMDMs in response to LPS exposure (Fig. 2e). Succinate sustains IL-1β production by promoting HIF-1α stabilization in pro-inflammatory macrophages^3,9^. Therefore, we envisioned that reduced succinate abundance imposed by mannose upon LPS exposure might underlie the impact of such sugar on BMDMs activation. To test this hypothesis, we assessed the effect of mannose on *Il1b* expression in macrophages cultured in a medium supplemented with succinate in the form of its cell-permeable analog diethyl-succinate^3,9^. As expected, diethyl-succinate increased LPS-induced *Il1b* expression in BMDMs and, importantly, repressed significantly the capability of mannose to impair *Il1b* expression, upon LPS exposure (Fig. 2f). In line with such changes, diethyl-succinate rescued the levels of succinate in LPS-stimulated BMDMs treated with mannose (Supplementary Fig. 4F) but did not elicit significant changes in steady-state levels of fumarate and α-ketoglutarate (Supplementary Fig. 4F), two TCA cycle metabolites playing a role in regulating macrophage response to LPS^24^, thus indicating that the impact of mannose on succinate accumulation is instrumental for opposing macrophage activation. This result prompted us to investigate the effect of mannose on succinate-dependent HIF-1α activation in response to LPS. In line with the effects on succinate accumulation, mannose treatment decreased HIF-1α levels, measured by western blotting, in mouse and human derived macrophages activated by LPS in vitro (Fig. 2g and Supplementary Fig. 5A) as well as in peritoneal macrophages isolated from mice challenged with LPS (Supplementary Fig. 5B). The effects of mannose treatment on HIF-1α activation was abrogated when BMDMs were incubated with diethyl-succinate (Supplementary Fig. 5C), indicating that mannose impairs LPS-induced HIF-1α activation mainly by decreasing succinate abundance in macrophages. Next, we determined the extent to which the impairment of HIF-1α activation contributes to mannose-mediated suppression of *Il1b* expression. For this aim *Hif1a* expression was silenced by transducing BMDMs with two independent shRNAs (Supplementary Fig. 5D). As expected, decreased *Il1b* expression was detected in HIF-1α-silenced macrophages, with respect to HIF-1α-expressing counterparts, upon LPS exposure (Supplementary Fig. 5E). Importantly, *Hif1a* silencing abolished the ability of mannose to further affect *Il1b* expression (Supplementary Fig. 5E), indicating that the impairment of HIF-1α activation is a response to mannose instrumental for dampening *Il1b* expression. Overall, these data demonstrate that mannose decreases glucose catabolism in macrophages and impairs succinate-mediated HIF-1α activation, thus reducing LPS-induced *Il1b* expression (Fig. 2h).

### MPI dictates response of LPS-activated macrophages to mannose

**Fig. 3:**
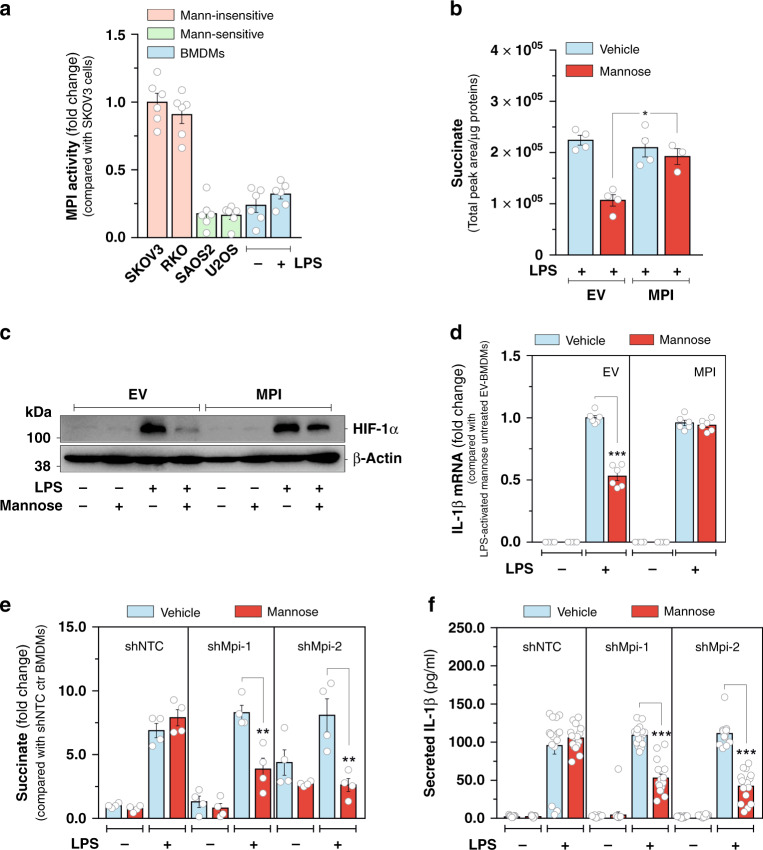
MPI activity dictates response of LPS-activated macrophages to mannose. **a** Measurement of MPI enzymatic activity in the indicated cell lines and in BMDMs cultured in the presence or absence of LPS for 24 h. Data are presented as mean ± s.e.m. of two independent experiments performed in technical triplicate, pooled together. **b** Effects of MPI overexpression in BMDMs on the intracellular levels of succinate measured after 24 h of LPS stimulation in the presence or absence of 11 mM U-^13^C_6_-Mannose in the culture medium. The sum of all the isotopologues was used to calculate the total abundance of the metabolite. Data are presented as mean ± s.e.m. of two independent experiments performed in technical duplicate, pooled together. **c** Representative image of HIF-1α, of an experiment performed once, of BMDMs cultured for 24 h in RPMI medium containing 11 mM glucose in the presence or absence of 11 mM mannose. β-Actin was used as a loading control. **d** Effect of MPI overexpression on IL-1β mRNA levels measured in resting and LPS-activated BMDMs cultured as in **c**. Data are presented as mean ± s.e.m. of two independent experiments performed in technical triplicate, pooled together. **e** Effect of MPI silencing on intracellular succinate abundance measured in resting and LPS-activated BMDMs cultured for 24 h in RPMI medium containing 11 mM glucose in the presence or absence of 4 mM mannose. Data are presented as mean ± s.e.m. of two independent experiments performed in technical duplicate pooled together. **f** Effect of MPI silencing on IL-1β secreted in media of resting and LPS-activated BMDMs cultured as in **e**. Data are presented as mean ± s.e.m. of *n* = 12 wells pooled from two independent experiments. In **b** and **d**–**f**, *P < 0.005; **P < 0.01; ***P < 0.001 (two-tailed Student’s *t*-test). Source data are provided as a Source Data file.

Next we explored the molecular determinants of macrophage sensitivity to mannose. We have recently demonstrated that MPI, apical enzyme in the mannose catabolic pathway (Fig. 2h), opposes the anti-tumor effects of such sugar, by preventing mannose-6-phosphate accumulation in cancer cells^21^. Prompted by this knowledge, we measured MPI enzymatic activity in cultured macrophages. We found that it did not change substantially in BMDMs in response to LPS treatment (Fig. 3a). However, its extent, measured both in resting and LPS-activated BMDMs, was much lower than that determined in previously reported mannose-insensitive cell lines (SKOV3 and RKO) and comparable to that measured in mannose-sensitive ones (SAOS2 and U2OS)^21^ (Fig. 3a), suggesting a pivotal role for MPI in priming macrophages to the anti-inflammatory effect of mannose. To test this hypothesis, BMDMs were transduced with a lentiviral vector expressing mouse *Mpi* cDNA (Supplementary Fig. 6A) and metabolically assessed upon LPS exposure. As expected, MPI overexpression decreased mannose-6-phosphate levels accumulating in LPS-activated BMDMs upon U-^13^C_6_-mannose incubation compared with the empty-vector transduced counterparts (Supplementary Fig. 6B). As a result of mannose-6-phosphate clearance, increased MPI levels abrogated the impact of mannose on glucose utilization in LPS-activated BMDMs, as indicated by the significant restoration of intracellular glucose-6-phospate abundance (Supplementary Fig. 6C) as well as both overall and glucose-derived lactate (^12^C-lactate) levels (Supplementary Fig. 6D), used as proxy for glycolytic activity in macrophages. Importantly, MPI overexpression rescued also the effect of LPS on succinate accumulation in mannose-treated BMDMs (Fig. 3b). Consistent with this, restoration of both LPS-induced HIF-1α activation (Fig. 3c) and *Il1b* gene expression (Fig. 3d) were observed in MPI-transduced BMDMs treated with mannose, compared with empty-vector-transduced counterparts. To corroborate the role of MPI in determining sensitivity of macrophages to mannose, we silenced *Mpi* expression by transducing BMDMs with two independent shRNAs (Supplementary Fig. 6E). Strikingly, suppression of *Mpi* gene expression potentiated the impact of sub-effective doses of mannose on intracellular accumulation of lactate (Supplementary Fig. 6F) and succinate (Fig. 3e) as well as IL-1β production induced by LPS exposure in BMDMs (Fig. 3f). Furthermore, contrarily to MPI silencing, overexpression of phosphomannomutase, achieved by transducing BMDMs with a lentiviral vector expressing mouse *Pmm2* cDNA (Supplementary Fig. 6F), did not enhance the impact of sub-effective mannose concentrations on IL-1β production in LPS-activated macrophages (Supplementary Fig. 6H). Overall, such results indicate that, contrarily to the phosphomannomutase-regulated pathway, MPI plays an instrumental role in determining response of pro-inflammatory macrophages to mannose.

### Mannose protects mice from DSS-induced ulcerative colitis

**Fig. 4:**
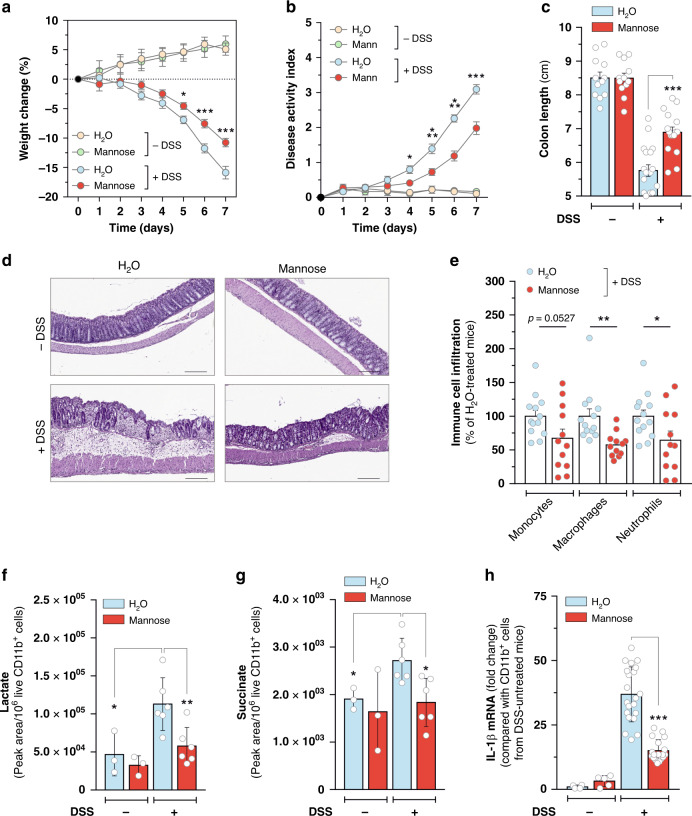
Mannose protects mice from DSS-induced ulcerative colitis. **a** Seven-week-aged C57BL/6 mice were pre-treated for 2 weeks with or without 20% (w/v) mannose given in drinking water. At the end of the pre-treatment, 3% (w/v) DSS in drinking water was administered ad libitum over a 7-day period. During DSS administration, mice were treated with 200 µl of either water or 20% (w/v) mannose by oral gavage twice a day. Body weights were determined daily and compared to the weights at the start of DSS treatment for each mouse. **b** Effect of mannose on disease activity index measured daily in mice described in **a** during DSS treatment schedule. In **a**, **b**, *P < 0.05; ***P < 0.001 (two-tailed Student’s *t*-test) compared with mannose-untreated DSS-administered mice. **c** Colon length of mi**c**e described in **a**. In **a**–**c** data are presented as mean ± s.e.m. of at least *n* = 16 (DSS-treated) and *n* = 12 (DSS-untreated) mice pooled from two independent experiments. **d** Representative H&E-stained images, out of three independently acquired, of colon sections from the indicated groups of mice treated as in **a** at the end of DSS administration (scale bar 200 µm). **e** FACS-mediated analysis of CD11b^+^ immune cell types infiltrating colon lamina propria of DSS-administered mice in response to mannose treatment, as described in **a**, at the end of the experiment. Data are presented as mean ± s.e.m. of *n* = 13 (H_2_O-treated) and *n* = 12 (mannose-treated) mice from two independent experiments. LC-MS-mediated analysis of lactate (**f**) and succinate (**g**) levels in CD11b^+^ cells isolated from colon lamina propria of mice treated as in **a** at the end of DSS administration. Data are presented as mean ± s.d. of *n* = 6 (DSS-treated) and *n* = 3 (DSS-untreated) mice. **h** qPCR analysis of IL-1β mRNA levels in CD11b^+^ cells isolated from colon lamina propria of mice treated as in **a** at the end of DSS administration. Data are presented as mean ± s.d. of *n* = 7 (DSS-challenged vehicle-treated), *n* = 8 (DSS-challenged mannose-treated) assessed in technical triplicate each and and *n* = 2 (DSS-untreated) mice assessed at least in technical duplicate each. In **c**, **e**–**h**, *P < 0.05; **P < 0.01; ***P < 0.001 (two-tailed Student’s *t*-test). Source data are provided as a Source Data file.

Aberrant activation of pro-inflammatory myeloid cells and sustained production of inflammatory cytokines, such as IL-1β, play instrumental role in the pathogenesis of inflammatory bowel diseases, such as ulcerative colitis and Crohn’s Disease^25–28^. Therefore, to further explore the impact of mannose on inflammatory response in vivo, we evaluated the preventive effects of such sugar on the progression of colitis induced by dextran sulfate sodium (DSS) in mice. Administration of supraphysiological doses of mannose via oral gavage had no major impacts on healthy mice. However, it opposed loss of body weight induced by DSS treatment (Fig. 4a) and delayed colitis progression (Fig. 4b), as indicated by the improvement of stools consistency (Supplementary Fig. 7A), attenuation of colorectal bleeding (Supplementary Fig. 7B) and counteraction of colon shortening (Fig. 4c and Supplementary Fig. 7C). In line with these data, qualitative (Fig. 4d) and quantitative (Supplementary Fig. 7D) histological examination of colon morphology revealed a marked decrease in the extent of epithelial disruption, crypt damage, and submucosal infiltration of inflammatory cells in colon of DSS-treated mice in response to mannose administration. In support of these observations, higher accumulation of myeloid (CD11b^+^) cells was detected by flow cytometry in colon lamina propria (LP) of DSS-treated mice compared with mannose-treated counterparts (Fig. 4e and Supplementary Fig. 8A). In contrast, colitis amelioration was not associated with changes in number of immunosuppressive T_regs_ accumulating in mice colon in response to DSS exposure (Supplementary Fig. 7E and Supplementary Fig. 8B). In addition to the preventive administration schedule, a delayed mannose treatment regimen (Supplementary Fig. 9A) was found to offer some anti-colitis therapeutic efficacy, as well. Indeed, oral administration of mannose after the onset of clinical signs of chemical-induced colitis, such as production of soft stools or the appearance of occult blood in feces, resulted in diminished proportions of DSS-treated mice showing severe colorectal bleeding and diarrhea compared with vehicle-treated counterparts (Supplementary Fig. 9B). Furthermore, mannose-treated mice showed longer colon length (Supplementary Fig. 9C) as well as decreased extent of colonic tissue damage and submucosal immune cell infiltration (Supplementary Fig. 9D) compared with the vehicle-treated group in response to DSS challenge.

Results obtained from such experiments prompted us to investigate the effects of mannose administration on metabolic activation of CD11b^+^ cells immunomagnetically isolated from colon submucosa of DSS-treated mice. Similarly to the impact on LPS-activated macrophages in vitro, mannose attenuated lactate (Fig. 4f) and succinate (Fig. 4g) levels increasing in CD11b^+^ cells infiltrating mice colon in response to DSS exposure. In line with such metabolic changes, lower *Il1b* expression was detected in such cells with respect to the myeloid counterparts isolated from the DSS-treated control group (Fig. 4h). It is noteworthy that mannose dampened the levels of HIF-1α increasing in CD11b^+^ cells in response to DSS challenge (Supplementary Fig. 7F), without eliciting major changes on its abundance in intestinal epithelial barrier cells of colitic mice (Supplementary Fig. 7G). Overall, these data indicate that mannose opposes metabolic changes supporting the engagement of pro-inflammatory signaling in colonic myeloid cells, resulting in colitis amelioration.

## Discussion

Macrophages are a main component of innate immunity playing a crucial role in promotion of inflammation in defence against pathogens. However, continuous production of pro-inflammatory mediators, resulting either from severe systemic reaction to infection (sepsis) or from unresolved responses to chronic tissue-damaging conditions, as those occurring in inflammatory bowel diseases, might be harmful and even lethal. Changes in nutrients utilization and metabolic fluxes drive malignant transformation and play instrumental roles in dictating cell fate and effector functions in immunity^1,29^. Therefore, interventions altering metabolic reprogramming in cancer as well as immune cells might offer valuable therapeutic options in both cancer and immunopathology^8,24^. Glucose is an essential nutrient both for neoplastic cells and pro-inflammatory macrophages. However, whereas glucose carbons are required for generation of novel biomass supporting proliferation of cancer cells, Warburg-like enhancement of glucose metabolism drives production of inflammatory cytokines, such as IL-1β, in classically activated macrophages^2,4,30^. In line with this, the hexokinase inhibitor 2-DG is known to impair LPS-induced IL-1β production in BMDMs^3^. Similarly, inhibition of glycolytic enzymes, such as glyceraldehyde-3-phosphate dehydrogenase and aldolase A, substantially accounts for the anti-inflammatory effects elicited by the immunosuppressive metabolite itaconate^31,32^ and synthetic analogs of fumarate in macrophages^6^. Overall, these findings clearly emphasized the therapeutic value of targeting glucose metabolism for successful treatment of inflammatory diseases.

Whereas the role of glucose in cancer and immune cells has been extensively investigated, the impact of different sugars on tumor growth and control of immune functions remains largely unexplored. We have recently demonstrated that the monosaccharide mannose inhibits growth of several forms of tumors by antagonizing glucose utilization of cancer cells^6^. Here, we report that mannose opposes classical macrophages activation by impairing LPS-induced *Il1b* gene expression. By employing stable isotope labeling techniques, we found that such phenotypic effect results from a diminished glucose metabolism in macrophages and a decreased LPS-induced succinate accumulation. Succinate is a prototypical inflammatory TCA cycle metabolite accumulating in pro-inflammatory macrophages to drive IL-1β production, by stabilizing the *Il1b* gene transcription factor HIF-1α^3,4^. Importantly, glycolysis inhibition is known to decrease succinate levels and to impair HIF-1α-dependent *Il1b* expression in LPS-activated macrophages^3^. Similarly, by silencing HIF-1α levels in BMDMs, here we provide evidence for the occurrence of an epistatic relationship between suppression of LPS-induced HIF-1α activation and impairment of *Il1b* expression in mannose-treated macrophages. Furthermore, rescuing both HIF-1α activation and *Il1b* expression, by supplementing mannose-treated BMDMs with a cell-permeable form of succinate, we demonstrated that the impact of mannose on succinate pool of LPS-activated macrophages is instrumental for impairing HIF-1α activation and optimal *Il1b* expression. Importantly, in line with the suppression of macrophage activation, pharmacological inhibition of glycolysis and genetic ablation of HIF-1α in myeloid cells are known to offer protection against septic shock in mice^3,4,33^. Likewise, here we demonstrate that administration of mannose decreases serum levels of IL-1β, accumulation of HIF-1α in peritoneal macrophages, and lethality in endotoxemic mice.

Mannose is a natural C-2 epimer of glucose. It is internalized in cells through GLUT transporters and then phosphorylated to mannose-6-phosphate by hexokinases. A minor fraction of such metabolite can be directed into glycosylation pathways; the majority of mannose-6-phosphate pool is catabolized into glycolysis after conversion to fructose-6-phophate in a reaction catalyzed by MPI^18^. We have recently demonstrated that cancer cells expressing low MPI levels inefficiently catabolizes mannose-6-phosphate which, accumulating intracellularly, inhibits glucose oxidation^21^. Similarly, here we show that mannose-6-phosphate levels increase in BMDMs in response to mannose and LPS treatments, resulting in a concomitant impairment of glucose catabolism. Importantly, we identified MPI as the molecular determinant dictating sensitivity of macrophages to such sugar. Indeed, MPI activity in BMDMs was found of comparable extent to that measured in previously identified mannose-sensitive cancer cells^21^. Furthermore, MPI overexpression totally abolished the impact of mannose on glucose utilization and rescued the engagement of HIF-1α signaling pathway supporting *Il1b* gene expression in LPS-activated BMDMs. Suppression of *MPI* expression potentiates the anti-proliferative effects of mannose on cancer cells^21^. Similarly, we found that *Mpi* silencing in BMDMs enhanced the impact of sub-effective mannose concentrations on LPS-induced IL-1β production. Importantly, beyond contributing to further understanding the mechanism of action underlying the anti-inflammatory effect of mannose, such in vitro observation might pave the way for preclinical testing of pharmacological MPI inhibitors in combination with mannose for potential treatment of inflammatory diseases.

Mannose was demonstrated to induce the generation of T_regs_ from naive CD4^+^ T cells. This effect underlies the capability of orally given mannose to suppress T cell-mediated immunopathology in mice, such as autoimmune type 1 diabetes and antigen-specific lung airway inflammation^20^. Here, we provide evidence for the utilization of mannose to ameliorate ulcerative colitis in mice. DSS-induced colitis has been extensively used as a preclinical model for investigating the instrumental contribution of myeloid cell activation to the progression of lethal intestinal inflammation in mice, in order to identify effective therapeutic strategies against inflammatory bowel diseases in humans^34^. We found that mannose decreased the number and activation of pro-inflammatory myeloid cells infiltrating colons of DSS-treated mice and to oppose HIF-1α activation, consistent with the role of myeloid HIF-1α activation in promoting DSS-induced colitis progression^35,36^. However, although similar treatment regimens were effective in increasing the frequency of immunosuppressive T_regs_ in lymphoid organs and pancreases of autoimmune diabetic mice^20^, mannose-induced colitis amelioration was not associated with changes in number of T_regs_ infiltrating mice colons in response to DSS challenge (Supplementary Fig. 7E). Importantly, analogously to the metabolic effects observed in LPS-activated macrophages cultured in vitro, we found that mannose treatment was effective in opposing the accumulation of lactate and succinate, supporting pro-inflammatory HIF-1α signaling activation in CD11b^+^-infiltrating colons of DSS-treated mice. Beyond providing mechanistic understanding of the anticolitic effects of mannose in vivo, our data support the potential utilization of glycolytic inhibitors (i.e. 2-DG) having metabolic effects similar to mannose on inflammatory myeloid cells, in order to counteract progression of ulcerative colitis. Furthermore, it is noteworthy that changes in colon microbiota contribute to colitis pathogenesis^37,38^. Mannose has been recently demonstrated to counteract high-fat diet-induced obesity in mice by altering gut microbial composition^39^. Therefore, future investigations, beyond the aim of the present work, are foreseen to determine whether, and the extent to which, restoration of colon dysbiosis, induced by mannose administration in mice, might contribute to colitis amelioration.

In summary, this study has uncovered, functionally and mechanistically, an unrecognized impact of sugar metabolism on pro-inflammatory macrophage activation, paving the way for a novel, safe and effective metabolic intervention against intestinal inflammation.

## Methods

### Cell lines and reagents

HEK293T, SKOV3, RKO, SAOS2, and U2OS cells (from ATCC) were maintained in DMEM (10313021 Thermo Fisher Scientific) containing 25 mM glucose supplemented with 10% FBS and 2 mM l-glutamine. Sources of reagents used in this study are indicated throughout the following sections. All reagents not described here were obtained from Sigma-Aldrich.

### Generation of BMDMs

If not otherwise stated throughout the main text or in each figure caption, bone marrow (BM)-derived macrophages (BMDMs) were prepared from 7- to 9-week-old CO_2_-euthanized C57BL/6N female mice and subjected to red blood cell lysis with ACK lysis buffer prior to neutralization in phosphate-buffered saline (PBS). BM was seeded at 3 × 10^5^ cells/well in six-well tissue-culture plates and differentiated in IMDM medium (Thermo Fisher Scientific) containing 25 mM glucose supplemented with 10% FBS (Gibco), 2 mM l-l-glutamine (Thermo Fisher Scientific) in the presence of 20 ng/ml recombinant mouse M-CSF (576404, Biolegend) for 7 days. When BMDMs from Balb/c background were used, they were generated analogously to C57BL/6N ones.

### Generation of human macrophages from peripheral blood monocytes

Peripheral blood mononuclear cells (PBMCs) were isolated from blood samples of healthy donors centrifuged on a FICOLL (Histopaque®-1077, Sigma-Aldrich 10771) density gradient at 400*g* for 40 min. CD14^+^ monocytes were then purified from the PBMCs by magnetic-activated cell sorting using human CD14 MicroBeads (130-050-201, Miltenyi Biotech). CD14^+^ monocytes were seeded at 3 × 10^5^ cells/well in six-well tissue-culture plates and differentiated in DMEM medium (D6546, Thermo Fisher Scientific) containing 25 mM glucose supplemented with 10% FBS (Gibco), 2 mM l-l-glutamine (Thermo Fisher Scientific) in the presence of 50 ng/ml recombinant human M-CSF (Biolegend) for 7 days. Human peripheral blood was collected upon informed consent from healthy volunteers according to the Institutional Ethical Committee approved protocol (TIGET09) and with the Declaration of Helsinki.

### Treatments

At the end of the differentiation, BMDMs and human macrophages were washed with PBS and used for in vitro treatments performed, respectively, in RPMI 1640 (R0883, Thermo Fisher Scientific, containing 11 mM glucose), DMEM (D5921, Thermo Fisher Scientific, containing 5.5 mM glucose) media, both supplemented with 10% FBS and 2 mM l-glutamine (Thermo Fisher Scientific). RAW 264.7 cells (from ATCC) were treated in DMEM medium (D5671, Thermo Fisher Scientific, containing 25 mM glucose) supplemented with 10% FBS and 2 mM l-glutamine. BMDMs, human macrophages, and RAW 264.7 cells were activated with 100 ng/ml LPS (Enzo Life Sciences) diluted in water for 24 h. Where used in vitro, d-mannose (M8574, Sigma-Aldrich) was solubilized in water and added at concentrations equimolar to that of glucose present in culture media, 2 h before LPS treatment, if not otherwise indicated. As control, mannose and/or LPS-untreated cells were incubated with corresponding volumes of water (vehicle). Diethyl-succinate and methyl-pyruvate were used at the concentrations indicated in each figure caption and incubated 2 h before LPS treatment.

### Measurement of cell viability

Cell viability was determined by 0.4% Trypan Blue dye exclusion test executed by the LUNA-II^TM^ Automated Cell Counter (Logos Biosystems).

### RNA extraction and real-time qPCR

RNA was extracted by TRIzol™ Reagent (15596018, Thermo Fisher Scientific) according to the manufacturer’s instructions. For real-time qPCR analysis, 1 μg of total RNA was retro-transcribed into complementary DNA using SuperScript VILO reverse transcriptase (11755-050, Invitrogen). cDNAs were mixed with PowerUp™ SYBR™ Green Master Mix (A25742, Applied Biosystems) according to the manufacturer’s instructions and 4 pmol of both forward and reverse primers for detecting mRNA levels of mouse and human genes. A complete list of all primers used, including the names and sequences, is supplied as Supplementary Table 1. qPCR was carried out using a 7500 fast real-time PCR system (Applied Biosystems) and the amplification steps were 95 °C for 20 s, followed by 40 cycles of 95 °C for 3 s and 60 °C for 30 s. The relative quantification of each mRNA was carried out with the comparative threshold cycle method using ribosomal protein S18 (Rps18/RPS18) or β-actin (Actb) for normalization of in vitro and in vivo studies, respectively.

### Enzyme-linked immunosorbent assay (ELISA)

IL-1β concentrations in BMDMs and RAW 264.7 culture medium as well as mice serum was measured using IL-1 beta mouse-uncoated ELISA Kit (88701376, Invitrogen), according to the manufacturer’s instructions. For inflammasome activation, BMDMs were incubated with 2.5 mM ATP for 40 min after priming with LPS for 24 h. TNF and IL-6 levels were determined in BMDMs and RAW 264.7 culture medium using, respectively, TNF (88-7324-22 Invitrogen) and IL-6 mouse (88-7064-22) uncoated ELISA Kits according to the manufacturer’s instructions. Absorbances were measured at a wavelength of 450 nm, subtracting the values measured at 570 nm, using a microplate reader (Bio-Rad).

### Measurement of Caspase-1 activity

For inflammasome activation, 4.5 × 10^4^ BMDMs were incubated with 2.5 mM ATP for 40 min after priming with LPS for 24 h. Enzymatic activity of Caspase-1 was determined in BMDMs with the Caspase-Glo^®^ 1 Inflammasome Assay (Promega) according to the manufacturer’s instructions. Luminescence was recorded by the Victor Multilabel Plate Reader.

### Immunoblotting

Cells were washed twice with cold PBS and lysed in radioimmunoprecipitation assay (RIPA) buffer supplemented with protease (cOmplete™ Mini EDTA-free Protease, Roche) and phosphatase (PhosSTOP EASYpack, Roche) inhibitor cocktails. Protein concentration was determined with the BCA Protein Assay Kit (23227, Pierce) using BSA as a standard. Equal amounts of protein were mixed with reducing Laemmli 4× buffer, warmed at 95 °C for 5 min, and loaded on 8% gels for SDS-PAGE. After electrophoretic separation, proteins were blotted onto 0.45 mm nitrocellulose (Amersham Protran), blocked with 10% non-fat milk in Tris-buffered saline-Tween, and incubated at 4 °C overnight with the following antibodies: anti-HIF-1 alpha (rabbit polyclonal, Novus Biologicals NB100-499, 1:200 for mouse macrophages and mouse monoclonal, BD Biosciences 610958, 1:500 for human macrophages), anti-MPI (mouse monoclonal, Santa Cruz Biotechnology sc-393477, 1:500), anti-Vinculin (mouse monoclonal, Sigma-Aldrich SAB4200729, 1:5000), and anti-β-Actin (mouse monoclonal, Sigma-Aldrich, A5441, 1:10000). Membranes were then washed and incubated with HRP-linked anti-rabbit (NA934V, GE Healthcare) or anti-mouse (NA931V, GE Healthcare) secondary antibodies at 1:10,000 dilution. Chemiluminescent signal was acquired using a ChemiDoc^TM^ MP Imaging System (Bio-rad) after incubation with Supersignal West Pico PLUS Chemiluminescent Substrate (34580, Thermo Fisher Scientific).

### Metabolite extraction and LC-MS

For ^13^C-glucose-tracing assays, BMDMs were treated in glucose-free RPMI 1640 medium (11879, Thermo Fisher Scientific) containing 2 mM l-glutamine, supplemented with 10% FBS and 11 mM U-^13^C_6_-d-glucose (CLM-1396, Cambridge Isotopes Laboratories) in the presence or absence of 11 mM unlabeled d-mannose for 24 h. For ^13^C-mannose-tracing assays and extracellular (exo-) metabolites determination, BMDMs were treated in 10% FBS, 2 mM l-glutamine-supplemented RPMI 1640 medium (31870074, Thermo Fisher Scientific) containing 11 mM unlabeled d-glucose in the presence or absence of U-^13^C_6_-d-mannose (CLM-6567, Cambridge Isotopes Laboratories) for 24 h. At the end of the treatments, monolayers were rapidly washed three times with ice-cold PBS and extracted with 600 μl of ice-cold extraction solution, composed of methanol, acetonitrile, and water (5:3:2), for endo-metabolite determination. Alternatively, media were diluted 1:50 in extraction solution for exo-metabolites analyses. Media derived from wells lacking cells but incubated in the same conditions were used as a reference to quantify the exchange rate (consumption/secretion) of exo-metabolites. To measure mannose concentration in blood, mouse tail tipping was performed before the first intraperitoneal injection of 2 g/kg mannose and 15 min after each (*n* = 3) administration, given hourly. Blood samples were then diluted in 250 µl of extraction solution (1:50 dilution). Culture media, cells, and blood extracts were then centrifuged at 16,000*g* for 30 min at 4 °C and the supernatants were analyzed by liquid chromatography-mass spectrometry (LC-MS). Endo-metabolites and exchange rates were normalized to the protein content in each well determined, at the end of the experiment, using the Lowry assay.

For endo-metabolites measurements from stable (^13^C)-tracing and diethyl-succinate-rescue experiments as well as for blood mannose levels determination, an Exactive Orbitrap mass spectrometer (Thermo Fisher Scientific) was used together with a Thermo Fisher Scientific Accela HPLC system. The HPLC setup consisted of a ZIC-pHILIC column (SeQuant, 150 mm × 2.1 mm, 5 µm, Merck KGaA) with a ZIC-pHILIC guard column (SeQuant, 20 mm × 2.1 mm) and an initial mobile phase of 20% 20 mM ammonium carbonate, pH 9.4 and 80% acetonitrile. Metabolites extracts (5 µl) were injected and metabolites were separated over a 15-min mobile phase gradient, decreasing the acetonitrile content to 20%, at a flow rate of 200 μl/min and a column temperature of 45 °C. All metabolites were detected across a mass range of 75–1000*m*/*z* using the Exactive mass spectrometer at a resolution of 25,000 (at 200*m*/*z*), with electrospray ionization and polarity switching to enable both positive and negative ions to be determined in the same run over a total analysis time of 23 min. For blood mannose levels determination, longer runs of 37 min, a 30 min gradient with the same solvent was used, at a flow rate of 100μl/min and a column temperature of 30 °C, as previously described^21^. Lock masses were used and the mass accuracy obtained for all metabolites was below 5 p.p.m. Data were acquired with Thermo LCquan 2.7 (Thermo Fisher Scientific) software.

For determination of intracellular lactate and succinate levels in CD11b^+^ LP mononuclear cells (described further below), shRNAs-transduced BMDMs as well as for exo-metabolites measurement, a SCIEX TripleTOF5600+ mass spectrometer equipped with on-line UPLC Agilent 1290 was used. MS acquisition was done in negative polarity. Chromatographic separations occurred on a Sequant pZIC-HILIC (150×2.1 mm, Merck Millipore) capillary column, packed with 5-µm polymer with an HILIC SecurityGuard (ULTRA Cartridge UHPLC HILIC 2.1 mm, Phenomenex). A gradient of eluents A (acetonitrile) and B ((NH_4_)_2_CO_3_ 20 mM + 0.1% NH_4_OH) was used to achieve separation (200 µl/min flow rate), from 20% B to 80% B in 15 min; the column temperature was set at 45 °C, while the autosampler was set at 4 °C. Full scan spectra were acquired in the mass range from *m*/*z* 75 to 1000. The mass accuracy obtained for all metabolites was below 5 p.p.m. Acquired data were analyzed using MultiQuant Full 3.0.3 (SCIEX) software.

### Measurement of MPI activity

Enzymatic activity of MPI was determined spectrophotometrically in cultured BMDMs and each cell line tested by a coupled enzymatic reaction, as previously reported^21^. Briefly, cell pellets were lysed by three freezing/thawing cycles. Eighty micrograms of post-nuclear protein fractions were incubated in a buffer containing 200 mU of phosphoglucose isomerase, 500 mM glucose-6-phosphate dehydrogenase, 1 mM NADP+, 40 mM Tris-HCl, pH 7.4, 6 mM MgCl_2_, 5 mM Na_2_HPO_4_/KH_2_PO_4_. Reactions were started by addition of 1 mM mannose-6-phosphate, and the production of NADPH was assessed at room temperature by measuring the OD at 340 nm.

### shRNA transductions

The lentiviral non-targeted NTC shRNA and shRNA plasmids against *Mpi*, *Hif1a*, and *Mrc1* were purchased from Sigma-Aldrich and identified as follows: Mpi-1 shRNA, TRCN0000252480 (5′-CCGGAGATCCTTGACAACCGTATTTCTCGAGAAATACGGTTGTCAAGGATCTTTTTTG-3′); Mpi-2 shRNA, TRCN0000252478 (5′-CCGGAGTAAATTTGGCATTAGTAACCTCGAGGTTACTAATGCCAAATTTACTTTTTTG-3′); Hif1a-1 shRNA, TRCN0000232220 (5′-CCGGCCCATTCCTCATCCGTCAAATCTCGAGATTTGACGGATGAGGAATGGGTTTTTG-3′); Hif1a-2 shRNA, TRCN0000232222 (5′-AATTCAAAAATGGATAGCGATATGGTCAATGCTCGAGCATTGACCATATCGCTATCCA-3′); Mrc1-1 shRNA, TRCN0000054794 (5′-CCGGGCACCCATTTAATGTACCCATCTCGAGATGGGTACATTAAATGGGTGCTTTTT-3′); Mrc1-2 shRNA, TRCN0000054795 (5′-CCGGCCTCTGGTGAACGGAATGATTCTCGAGAATCATTCCGTTCACCAGAGGTTTTT-3′). Lentiviral plasmids were transfected into HEK293T cells together with packaging and envelope plasmids (psPAX2 and VSV-G) using the calcium phosphate procedure. Two days after transfection, the growth medium containing lentiviruses was filtered through a 0.45-μm pore filter, mixed with 4 µg/ml Polybrene (H9268, Sigma-Aldrich) and transferred to the recipient BMDMs. HEK293T cells were further cultured in fresh medium for 24 h. Next day, infection was repeated as above. After lentivirus infection, BMDMs were selected with 1 μg/ml Puromycin (Sigma-Aldrich, Cat#: P9620) for 48 h. Then, medium was changed and BMDMs treated as described above.

### Cloning and overexpression of mouse *Mpi* and *Pmm2*

The blasticidin S deaminase cassette was subcloned from the lentiCRISPR v2-Blast transfer vector (purchased from Addgene) into the *Rsr*II–*Nhe*I sites of a bidirectional miRNA reporter lentiviral vector (kindly provided by Dr. Simone Cenci, IRCCS San Raffaele Scientific Institute) described previously^40,41^. The newly generated transfer vector, named BSD1, was used for cloning either mouse *Mpi* or mouse *Pmm2* cDNAs. For this aim, RNA was extracted from mouse liver by the RNeasy Plus Mini Kit and retro-transcribed into complementary DNA by SuperScript™ II Reverse Transcriptase (18064014, Thermo fisher Scientific). Mouse *Mpi* and *Pmm2* cDNAs were amplified by Platinum™ PCR SuperMix High Fidelity (12532016, Thermo fisher Scientific) according to the manufacturer’s instructions using the following primers: *Mpi* (forward: 5′-GCGCCGACCGGTATGGCGAGTCCGCGAGT-3′; reverse: 5′-GGCGCCGTCGACCTACAGCAGACAGCAGGCCC-3′); *Pmm2* (forward: 5′-GCGCCGACCGGTATGGCCACTCTCTGTCTCTTCGAC-3′; reverse: 5′-GGCGCCGTCGACTCAAGGGAAGAGCCCCTCACAG-3′). Amplified mouse *Mpi* and *Pmm2* cDNAs were then cloned into the *Age*I–*Sal*I sites of BSD1 vector. Lentiviral stocks were prepared, concentrated, and titered as previously described^42^. Briefly, self-inactivating (SIN) LV vector were produced using either the empty or cDNA- cloned BSD1 transfer vector, the packaging plasmid pMDLg/pRRE, Rev-expressing pCMV-Rev, and the VSV-G envelop-encoding pMD2.VSV-G plasmids. BMDMs were transduced with a MOI = 5. Two days after the transduction, BMDMs were selected with 1 μg/ml Blasticidin S (R21001, Gibco) for 72 h. Then, medium was changed and BMDMs treated as described above .

### Induction and macroscopic assessment of colitis in mice

For the evaluation of protective (prophylactic) efficacy of mannose against colitis in mice, seven-week-aged C57BL/6N mice were pre-treated with or without 20% (w/v) d-mannose given in drinking water ad libitum for 2 weeks. Then, colitis was induced by administration of 3% dextran sodium sulfate (DSS) (36–50 kDa, colitis grade MP Biomedicals) in filter-purified drinking water ad libitum for 7 days. Throughout DSS administration, mice were treated with 200 µl of either water or 20% (w/v) mannose by oral gavage twice a day. The severity of colitis was assessed daily by recording body weights (expressed as the percent of change of the weight at the start of DSS treatment for each mouse). To assess macroscopic symptoms of inflammation, feces were collected daily from each mouse and evaluated for consistency and the presence of occult blood by Hemoccult SENSA (Beckman Coulter). The disease activity index^43^ (DAI) was then calculated as the average of three different parameters: body weight loss (score 1: 1–5%; score 2: 5–10%; score 3: 10–15%; score 4: more than 15%), blood in the stools (score 0: no blood; score 2: occult blood; score 4: visible anal bleeding), and stools consistency (score 0: well formed; score 2: soft; score 4: diarrhea). At the end of treatment (day 7 of DSS treatment), mice were killed, colons were excised, and their lengths, from the end of the cecum to the anus, were measured.

For the evaluation of therapeutic efficacy of mannose against DSS-induced colitis, 7-week-aged C57BL/6 mice were provided with 3% (w/v) DSS in drinking water ad libitum for 5 days and then returned to normal drinking water until the end of the experiment (day 10). Starting from day 3 of DSS administration—time sufficient to induce formation of either soft stools or the appearance of occult blood in feces—mice were treated with 200 µl of either water or 20% (w/v) mannose by oral gavage twice a day, until the end of the experiment. Macroscopic symptoms of inflammation and colon length were measured as described above.

### Histological assessment of colitis in mice and immunohistochemistry

Colons were collected from sacrificed mice at the end of each treatment schedule, as described above, fixed in 10% neutral-buffered formalin, and embedded in paraffin. Serial 5 µm microtome-cut sections were then stained with hematoxylin and eosin (Sigma-Aldrich). Slides were acquired with an Aperio AT2 digital scanner (Leica Biosystems) and analyzed with an Aperio Imagescope (Leica Biosystem). Scoring of colonic inflammation was determined as reported by Nagahama et al.^44^ with minor modifications, by assessing mucosa thickening, inflammatory cells, and submucosa cell infiltration. Each criterion was scored as 0–4, and the sum of each score was defined as the histological score.

Immunohistochemical staining of HIF-1α was performed on colon sections of mice with the anti-HIF-1 alpha antibody (Abcam ab2185, 1:2000) and antigen unmasking in citrate buffer. Sections were incubated with specific Detection KIT-HRP (Abcam, ab236469) according to the manufacturer’s instructions, then counter-stained with hematoxylin to visualize nuclei, dehydrated and mounted in DPX (Sigma-Aldrich). Bright-field images were acquired with an Aperio AT2 digital scanner (Leica Biosystems) and analyzed with an Imagescope (Leica Biosystem) or using a Zeiss Axio Imager M2m microscope with a ×10 or ×20 objective.

### FACS characterization of colon lamina propria-infiltrating immune cells

Colon lamina propria mononuclear cells (LPMCs) were obtained from healthy and inflamed colonic tissues, as previously described^44^. Briefly, at the end of DSS treatment, colons were cut longitudinally and washed in PBS containing 5% FBS and 2 mM EDTA for 10 min at 37 °C. Enzymatic digestion was then performed in RPMI 1640 medium supplemented with 10% fetal bovine serum, 0,25 mg/ml collagenase type VIII (Sigma-Aldrich), 20 µg/ml DNase I (Roche Diagnostics), 100 U/ml penicillin, and 100 mg/ml streptomycin for 45 min at 37 °C with gentle shaking. Then, cell suspension was washed with FACS buffer (PBS supplemented with 0.5% BSA and 1 mM EDTA) and centrifuged at 300*g* for 8 min, before being clarified over a 70-µm cell strainer.

To characterize the different myeloid populations, 1 × 10^6^ murine LPMCs were stained for 20 min at 4 °C with the following fluorochrome-conjugated antibodies (Biolegend) diluted 1:250 in FACS buffer: FITC-conjugated anti-mouse CD45 (clone 30-F11), APC-conjugated anti-mouse CD11c (clone N418), Pe-Cy7-conjugated anti-mouse/human CD11b clone (M1/70), Ly6C PerCPCy5.5-conjugated anti-mouse Ly6C (clone HK1.4), PE-conjugated anti-mouse F4/80 (clone BM8), APC-Cy7-conjugated anti-mouse Ly6g (clone 1A8), and brilliant violet 510-conjugated anti-mouse MHCII (clone M5/114.15.2). Cell populations were gated on DAPI-negative (live) single cells and identified as follows: monocytes (CD45^+^, CD11b^+^, CD11c^−^, F4/80^−^, Ly6C^+^), macrophages (CD45^+^, CD11b^+^, CD11c^−^, F4/80^+^, Ly6C^−^) and neutrophils (CD45^+^, CD11b^+^, CD11c^−^, F4/80^−^, Ly6G^+^, Ly6C^+^).

To assess infiltration of regulatory T cells (T_regs_, identified as CD45^+^, CD3^+^, CD4^+^, CD25^+^, Foxp3^+^ cells) 1 × 10^6^ murine LPMCs were washed in PBS and stained with Zombie Violet (Biolegend) for 20 min at 4 °C. Afterwards samples were stained with the following fl for 20 mi-conjugated antibodies (Biologend) diluted 1:250 in FACS buffer: FITC-conjugated anti-mouse CD45 (clone 30-F11), APC-conjugated anti-mouse CD25 (clone PC61), PerCPCy5.5-conjugated anti-mouse CD4 (GK1.5), and APC-Cy7-conjugated anti-mouse CD3e (clone 145-2C11). Cells were then fixed and permeabilized with BD Cytofix/Cytoperm Fixation/Permeabilization Solution Kit for 10 min at RT, according to the manufacturer’s protocol. Cells were then stained with Pe-Cy7-conjugated anti-mouse Foxp3 antibody (clone FJK-16s, eBioscience) diluted 1:250. To assess MRC1 levels in BMDMs transduced with lentiviral non-targeted shRNA and shRNA plasmids against *Mrc1*, cells were incubated with Aqua live/dead viability staining marker (Invitrogen, Carlsbad, US) and an Fc block reagent (purified anti-CD16/32, clone 93, eBioscience-Thermo Fisher, Waltham, US). Then, the following murine antibodies, diluted in PBS^−/−^ containing 2% FCS, 2 mM EDTA, and 0.05% NaN_3_, were used: anti-CD45-FITC (clone 30-F11), anti CD11b-Pacific Blue (clone M1/70), anti-CD11c-APC (clone N418), anti-F4/80-PE (clone BM8), and anti-CD206-PeCy7 (clone C068C2) from Biolegend (San Diego, US). All samples were acquired using BD FACSCanto II (BD Bioscience); data were analyzed with FlowJo software (Treestar).

### Immunomagnetic sorting and analysis of CD11b^+^ LPMCs

CD11b^+^ were isolated from LPMCs, obtained from healthy and inflamed colonic tissues, by magnetic cell sorting using CD11b microbeads (Miltenyi Biotec) according to the manufacturer’s instructions. RNA was extracted by the RNeasy Plus Mini Kit (74136, Qiagen) and used for real-time qPCR analyses as described above. The relative quantification of *Il1b* mRNA levels was carried out with the comparative threshold cycle method using β-actin (Actb) for normalization. For determination of lactate and succinate levels, 1 × 10^6^ live (trypan blue-negative) CD11b^+^ cells were incubated with 300 μl of ice-cold extraction solution and extracted metabolites measured by LC-MS as described above.

### Endotoxin-induced model of sepsis

Seven- to 9-week-aged male Balb/c mice were stimulated with a single intraperitoneal (i.p) injection of 20 mg/kg O55:B5 LPS diluted in saline solution and treated with 2 g/kg mannose, solubilized in saline solution, given hourly by i.p. injections for up to six times. LPS-untreated mice (when used) and mannose-untreated mice received corresponding volumes of saline solution, as control. Progression of disease was determined by two blinded investigators by monitoring mice every 2 h after LPS injection for clinical signs of endotoxic shock based on coat and eyes appearance, level of consciousness, locomotor activity, respiration rate, and quality scored between 0 and 4, as previously reported^44^. The humane end-point for the experiment was achieved when a combined score >12 or a maximal score in one individual parameter was totalized.

For IL-1β measurement and peritoneal macrophages isolation, control mice and counterparts stimulated with 20 mg/kg O55:B5 LPS were treated with either 2 g/kg mannose or saline control (vehicle) solution given hourly by i.p. injection. After 3 h LPS injection, mice were sacrificed in a CO_2_ chamber. Then, peritoneal macrophages were isolated as previously reported^45^, from which HIF-1α levels were determined by western blotting, as well as blood samples collected, for ELISA—determination of serum IL-1β levels, as described above.

### Ethical approval of animal studies

Male and female C57BL/6N and Balb/c mice were obtained from Charles River and bred in house. Animals were housed in individual ventilated cages in a barrier facility proactive in environmental enrichment under specific pathogen-free conditions in line with European Union regulations. All experimental animal procedures were approved by the Institutional Animal Committee of San Raffaele Scientific Institute.

### Statistics and reproducibility

Two-tailed Student *t*-tests were carried out with GraphPad Prism 7 software. When unequal variances between experimental groups were computed, Welch’s correction was applied. Potential outliers in Fig. 1c were identified by the ROUT method according to GraphPad guidelines and removed from experimental groups for more accurate calculation of statistical difference between means. For in vitro studies, statistical analyses were carried out using the number of wells as the sample size (*n*). Wells represent technical replicate samples setup and assessed under identical conditions in a single experiment. Details of the numbers of wells assessed and the number of independently replicated experiments are provided in each figure legend.

### Reporting Summary

Further information on research design is available in the Nature Research Reporting Summary linked to this article.

## Supplementary information

Supplementary Information

Reporting Summary

## Data Availability

All other data are available from the corresponding author upon reasonable requests. Source data are provided with this paper.

## Source data

Source Data

## References

[1] O’Neill LAJ, Kishton RJ, Rathmell J (2016). A guide to immunometabolism for immunologists. Nat. Rev. Immunol..

[2] Andrejeva G, Rathmell JC (2017). Similarities and distinctions of cancer and immune metabolism in inflammation and tumors. Cell Metab..

[3] Tannahill GM (2013). Succinate is an inflammatory signal that induces IL-1β through HIF-1α. Nature.

[4] Liu L (2016). Proinflammatory signal suppresses proliferation and shifts macrophage metabolism from Myc-dependent to HIF1α-dependent. Proc. Natl Acad. Sci. USA.

[5] Ip WKE, Hoshi N, Shouval DS, Snapper S, Medzhitov R (2017). Anti-inflammatory effect of IL-10 mediated by metabolic reprogramming of macrophages. Science.

[6] Kornberg MD (2018). Dimethyl fumarate targets GAPDH and aerobic glycolysis to modulate immunity. Science.

[7] Abboud G (2018). Inhibition of glycolysis reduces disease severity in an autoimmune model of rheumatoid arthritis. Front. Immunol..

[8] Patel CH, Leone RD, Horton MR, Powell JD (2019). Targeting metabolism to regulate immune responses in autoimmunity and cancer. Nat. Rev. Drug Discov..

[9] Mills EL (2016). Succinate dehydrogenase supports metabolic repurposing of mitochondria to drive inflammatory macrophages. Cell.

[10] Rodriguez AE (2019). Serine metabolism supports macrophage IL-1β production. Cell Metab..

[11] Yu W (2019). One-carbon metabolism supports S-adenosylmethionine and histone methylation to drive inflammatory macrophages. Mol. Cell.

[12] Ryan DG (2019). Coupling Krebs cycle metabolites to signalling in immunity and cancer. Nat. Metab..

[13] Murphy MP, O’Neill LAJ (2018). Krebs cycle reimagined: the emerging roles of succinate and itaconate as signal transducers. Cell.

[14] Littlewood-Evans A (2016). GPR91 senses extracellular succinate released from inflammatory macrophages and exacerbates rheumatoid arthritis. J. Exp. Med..

[15] Peruzzotti-Jametti L (2018). Macrophage-derived extracellular succinate licenses neural stem cells to suppress chronic neuroinflammation. Cell Stem Cell.

[16] Macias-Ceja DC (2019). Succinate receptor mediates intestinal inflammation and fibrosis. Mucosal Immunol..

[17] Peyssonnaux C (2007). Cutting edge: essential role of hypoxia inducible factor-1α in development of lipopolysaccharide-induced sepsis. J. Immunol..

[18] Sharma V, Ichikawa M, Freeze HH (2014). Mannose metabolism: more than meets the eye. Biochem. Biophys. Res. Commun..

[19] Kranjčec B, Papeš D, Altarac S (2014). D-mannose powder for prophylaxis of recurrent urinary tract infections in women: a randomized clinical trial. World J. Urol..

[20] Zhang D (2017). D-mannose induces regulatory T cells and suppresses immunopathology. Nat. Med..

[21] Gonzalez PS (2018). Mannose impairs tumour growth and enhances chemotherapy. Nature.

[22] Taylor PR (2005). Macrophage receptors and immune recognition. Annu. Rev. Immunol..

[23] DeRossi C (2006). Ablation of mouse phosphomannose isomerase (Mpi) causes mannose 6-phosphate accumulation, toxicity, and embryonic lethality. J. Biol. Chem..

[24] Scagliola, A., Mainini, F. & Cardaci, S. The TCA cycle at the crossroad between cancer and immunity. *Antioxid. Redox Signal*. 10.1089/ars.2019.7974 (2019). 10.1089/ars.2019.7974 31847530

[25] Neurath MF (2019). Targeting immune cell circuits and trafficking in inflammatory bowel disease. Nat. Immunol..

[26] Na YR, Stakenborg M, Seok SH, Matteoli G (2019). Macrophages in intestinal inflammation and resolution: a potential therapeutic target in IBD. Nat. Rev. Gastroenterol. Hepatol..

[27] Coccia M (2012). IL-1β mediates chronic intestinal inflammation by promoting the accumulation of IL-17A secreting innate lymphoid cells and CD4(+) Th17 cells. J. Exp. Med..

[28] Siegmund B, Lehr HA, Fantuzzi G, Dinarello CA (2001). IL-1β-converting enzyme (caspase-1) in intestinal inflammation. Proc. Natl Acad. Sci. USA.

[29] Vander Heiden MG, DeBerardinis RJ (2017). Understanding the intersections between metabolism and cancer biology. Cell.

[30] Nonnenmacher Y, Hiller K (2018). Biochemistry of proinflammatory macrophage activation. Cell. Mol. Life Sci..

[31] Qin W (2019). S-glycosylation-based cysteine profiling reveals regulation of glycolysis by itaconate. Nat. Chem. Biol..

[32] Liao, S. T. et al. 4-Octyl itaconate inhibits aerobic glycolysis by targeting GAPDH to exert anti-inflammatory effects. *Nat. Commun*. **10**, 5091 (2019). 10.1038/s41467-019-13078-5 PMC6841710 31704924

[33] Hu, K. et al. Caloric restriction mimetic 2-deoxyglucose alleviated inflammatory lung injury via suppressing nuclear pyruvate kinase M2-signal transducer and activator of transcription 3 pathway. *Front. Immunol*. **9**, 426 (2018). 10.3389/fimmu.2018.00426 PMC5840172 29552018

[34] Eichele DD, Kharbanda KK (2017). Dextran sodium sulfate colitis murine model: an indispensable tool for advancing our understanding of inflammatory bowel diseases pathogenesis. World J. Gastroenterol..

[35] Bäcker V, Cheung F-Y, Siveke JT, Fandrey J, Winning S (2017). Knockdown of myeloid cell hypoxia-inducible factor-1α ameliorates the acute pathology in DSS-induced colitis. PLoS ONE.

[36] Kim, Y. E. et al. HIF-1α activation in myeloid cells accelerates dextran sodium sulfate-induced colitis progression in mice. *Dis. Model. Mech*. **11**, dmm033241 (2018). 10.1242/dmm.033241 PMC6078398 29967068

[37] Manichanh C, Borruel N, Casellas F, Guarner F (2012). The gut microbiota in IBD. Nat. Rev. Gastroenterol. Hepatol..

[38] Zuo, T. & Ng, S. C. The gut microbiota in the pathogenesis and therapeutics of inflammatory bowel disease. *Front. Microbiol.* **9**, 2247 (2018). 10.3389/fmicb.2018.02247 PMC6167487 30319571

[39] Sharma V (2018). Mannose alters gut microbiome, prevents diet-induced obesity, and improves host metabolism. Cell Rep..

[40] Chiriaco M (2014). Dual-regulated lentiviral vector for gene therapy of X-linked chronic granulomatosis. Mol. Ther..

[41] Gentner, B. et al. Identification of hematopoietic stem cell-specific miRNAs enables gene therapy of globoid cell leukodystrophy. *Sci. Transl. Med*. **2**, 58ra84 (2010). 10.1126/scitranslmed.3001522 21084719

[42] Petrillo C (2018). Cyclosporine H overcomes innate immune restrictions to improve lentiviral transduction and gene editing in human hematopoietic stem cells. Cell Stem Cell.

[43] Genua M (2015). The urokinase plasminogen activator receptor (uPAR) controls macrophage phagocytosis in intestinal inflammation. Gut.

[44] Nagahama Y (2018). Regnase-1 controls colon epithelial regeneration via regulation of mTOR and purine metabolism. Proc. Natl Acad. Sci. USA.

[45] Czimmerer Z (2018). The transcription factor STAT6 mediates direct repression of inflammatory enhancers and limits activation of alternatively polarized macrophages. Immunity.

