# Supplementary material for: D-mannose suppresses macrophage IL-1β production
Source: Nat Commun. 2020 Dec 11;11:6343. doi: 10.1038/s41467-020-20164-6 (PMC7733482; doi:10.1038/s41467-020-20164-6)
Supplement: Supplementary file 1 — Supplementary Information [file 41467_2020_20164_MOESM1_ESM.pdf]

Supplementary Information

D-Mannose Suppresses Macrophage IL-1β Production

Torretta et al.

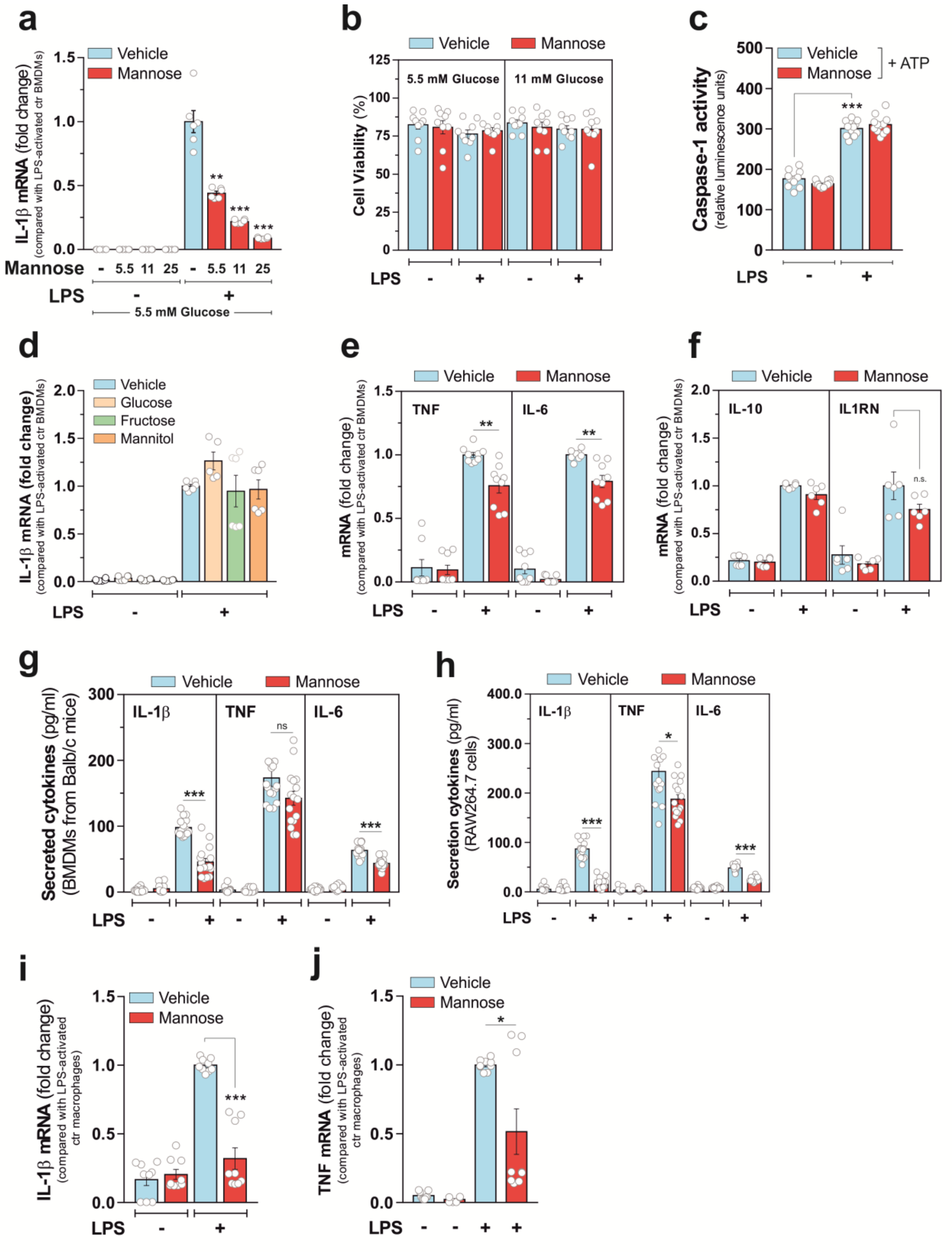

Supplementary Figure 1. Mannose impairs LPS-induced IL-1β production. (a) qPCR analysis of IL-1β mRNA levels in resting and LPS-activated BMDMs cultured for 24h in RPMI medium containing 5.5 mM glucose in the presence or absence of

the indicated concentrations of mannose. (**b**) Viability of BMDMs cultured in RPMI medium containing either 5.5 mM or 11 mM glucose in the presence or absence of corresponding equimolar concentrations of mannose. (**c**) Measurement of Caspase-1 activity in BMDMs cultured for 24h in RPMI medium containing 11 mM glucose in the presence or absence of 11 mM mannose. (**d**) IL-1β mRNA levels in BMDMs cultured for 24h in RPMI medium containing 11 mM glucose supplemented with additional 11 mM glucose, 11 mM fructose or 11 mM mannitol. qPCR analysis of IL-6 and TNF (**e**) as well as IL-10 and IL1RN (**f**) mRNA levels in BMDMs cultured for 24h in RPMI medium containing 11 mM glucose in the presence or absence of 11 mM mannose. (**g**) ELISA-mediated determination of IL-1β, TNF and IL-6 secreted in culture media of BMDMs generated from Balb/c mice treated as in (e). (**h**) ELISA-mediated determination of IL-1β, TNF and IL-6 secreted in culture media of RAW 264.7 cells cultured for 24h in DMEM medium containing 25 mM glucose in the presence or absence of 25 mM mannose. qPCR analysis of IL-1β (**i**) and TNF (**j**) mRNA levels in human macrophages cultured for 24h in DMEM medium containing 5.5 mM glucose in the presence or absence of 5.5 mM mannose. Data are presented as mean±s.e.m of: (a) n=6 wells pooled from two independent experiments performed in technical triplicate; (b) n=9 wells pooled from three independent experiments; (c) n=12 wells pooled from two independent experiments; (d) two independent experiments performed at least in technical duplicate, pooled together; three (e) and two (f) independent experiments performed in technical triplicate; (g) n=16 wells pooled from two independent experiments; (h) n=16 wells pooled from two independent experiments; (i-j) n=9 wells pooled from three independent experiments. * = *P* <0.05; ** = *P* <0.01; *** = *P* <0.001 (two-tailed Student's t-test); ns, not significant. Source data are provided as a Source Data file.

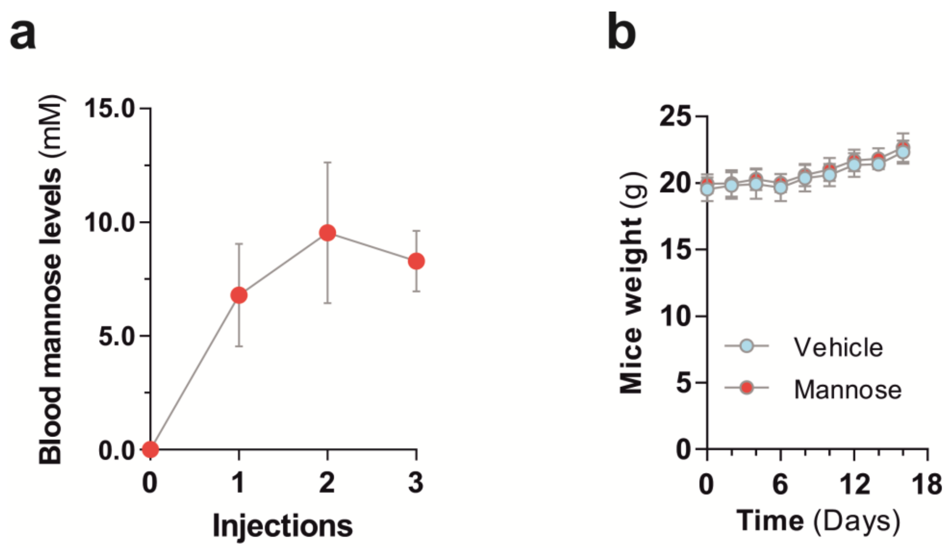

Supplementary Figure 2. Effects of mannose administration on body weight and mannose blood levels in mice. (**a**) Concentration of mannose in blood of n=3 mice determined before the first intraperitoneal injection of 2 g/kg mannose and 15 min after each (n=3) administration, given hourly. Data are presented as mean±s.d. of one experiment. (**b**) Body weights of n=6 mice per group treated each with either 2 g/kg mannose or saline control (vehicle) solution, given hourly by intraperitoneal injections six times a day. Data are presented as mean±s.d. of one experiment. Source data are provided as a Source Data file.

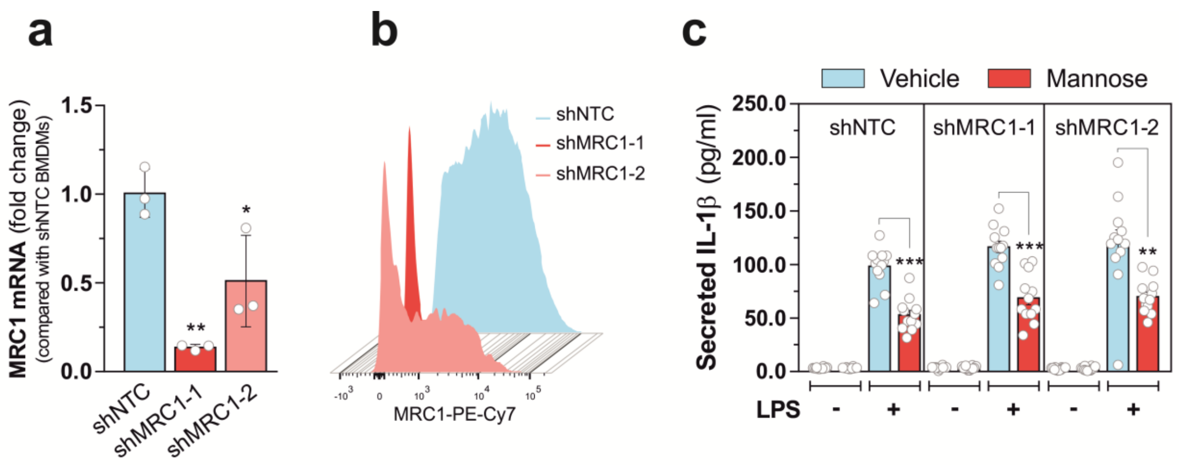

Supplementary Figure 3. Effects of MRC1 silencing on response of LPS-activated macrophages to mannose. (**a**) qPCR analysis of MRC1 mRNA levels in BMDMs transduced with lentiviral vectors expressing either a non-targeting control (shNTC) or two different shRNA sequences against mRNA of *Mrc1* gene (shMRC1-1 and shMRC1-1). Data are presented as mean±s.d. of one representative experiment performed in technical triplicate. * = *P* <0.05; ** = *P* <0.01; (two-tailed Student's t-test) compared to shNTC BMDMs. (**b**) FACS-mediated measurement of plasmamembrane MRC1 abundance in BMDMs treated as in (a) (representative image). (**c**) Effects of MRC1 silencing in BMDMs on IL-1β secretion in response to LPS. Data are presented as mean±s.e.m. of n=12 wells pooled from two independent experiments. ** = *P* <0.01; *** = *P* <0.001; (two-tailed Student's t-test). Source data are provided as a Source Data file.

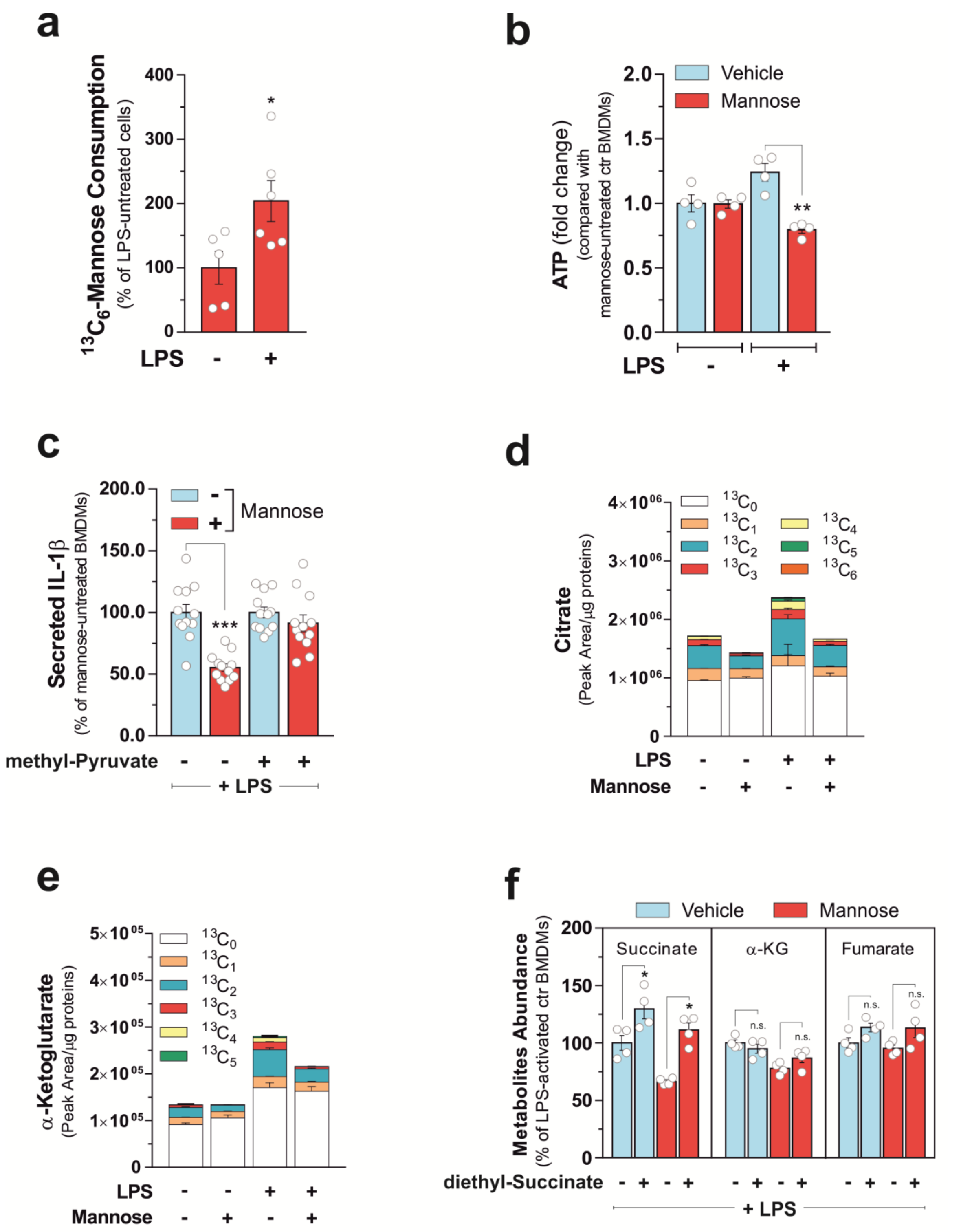

Supplementary Figure 4. Mannose impairs glucose catabolism and succinate production in LPS-activated BMDMs. (**a**) Mannose consumption rate in resting and LPS-activated BMDMs cultured for 24h in RPMI medium containing 11 mM glucose in the presence of 11mM U-$^{13}C_6$-Mannose Data are presented as

mean±s.e.m of n=6 wells pooled from two independent experiments. * = *P* <0.05 (two-tailed Student's t-test). (**b**) Intracellular levels of ATP in resting and LPS-activated BMDMs cultured for 24h in RPMI medium containing 11 mM glucose in the presence or absence of 11 mM mannose. Data are presented as mean±s.e.m of n=4 wells from two independent experiments. ** = *P* <0.01 (two-tailed Student's t-test). (**c**) ELISA-mediated determination of IL-1β secreted in culture media of LPS-activated BMDMs treated as in (b) in the presence or absence of 5 mM methyl-pyruvate in the medium. Data are presented as mean±s.e.m of n=12 wells pooled from two independent experiments. *** = *P* <0.001; (two-tailed Student's t-test). Intracellular levels of citrate (**d**) and α-ketoglutarate (**e**) in resting and LPS-activated BMDMs cultured for 24h in RPMI medium containing 11 mM U-$^{13}C_6$-Glucose in the presence or absence of 11 mM mannose. For each metabolite, the abundance of all the isotopologues is reported. Data are presented as mean±s.e.m of n=4 wells from two independent experiments. (**f**) Intracellular levels of the indicated metabolites measured in LPS-activated BMDMs treated as in (b) in the presence or absence of 5 mM diethyl-Succinate in the medium. Data are presented as mean±s.e.m of n=4 wells pooled from two independent experiments. *= *P* <0.05; (two-tailed Student's t-test); n.s., not significant. Source data are provided as a Source Data file.

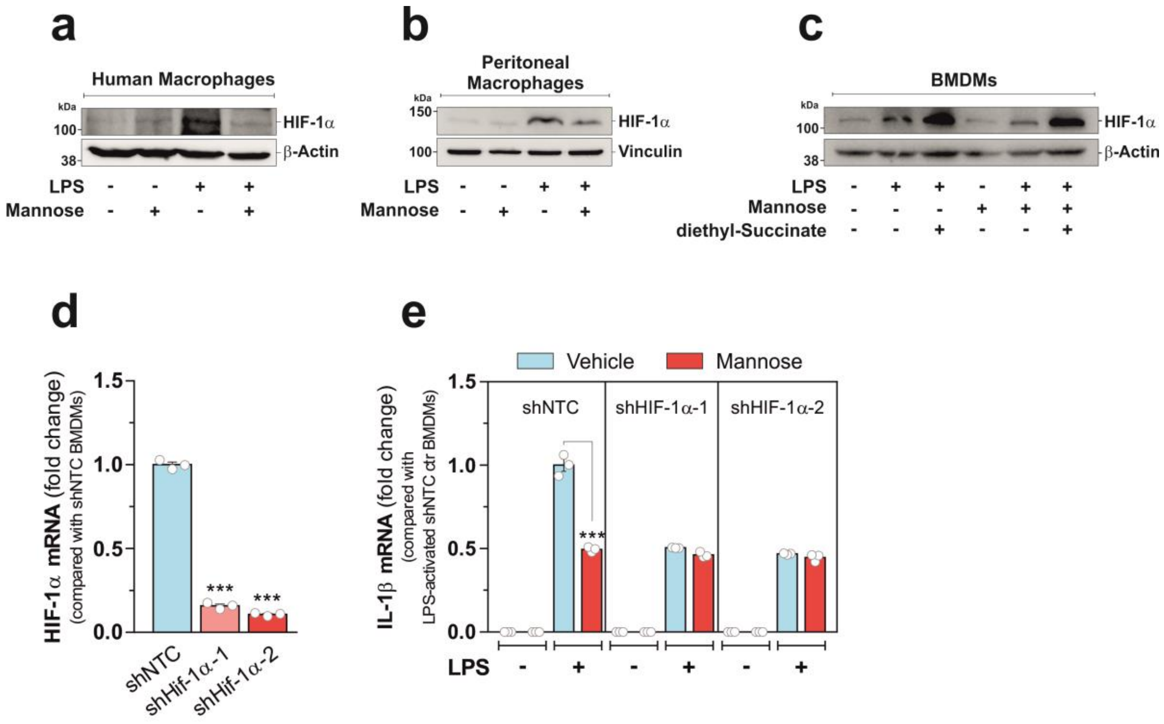

Supplementary Figure 5. Mannose suppresses succinate-mediated HIF-1α activation in LPS-stimulated macrophages. (**a**) Representative image of HIF-1α levels of an experiment performed once, measured by Western Blotting, in resting and LPS-activated human macrophages, differentiated from CD14$^+$ peripheral blood monocytes, cultured for 24h in DMEM medium containing 5.5 mM glucose in the presence or absence of 5.5 mM mannose. β-Actin was used as a loading control. (**b**) Representative image of HIF-1α levels of an experiment performed once, measured by Western Blotting, in peritoneal macrophages pooled from n=6 control mice and n=8 mice stimulated with 20 mg/kg O55:B5 LPS for 3h, treated with either 2 g/kg mannose or saline control (vehicle) solution given hourly by i.p. injection. Vinculin was used as a loading control. (**c**) Representative image of HIF-1α levels of an experiment performed once, measured by Western Blotting, in resting and LPS-activated BMDMs cultured for 24h in medium containing in 11mM glucose in the presence or absence of 11 mM mannose and 5 mM diethyl-Succinate, as indicated.

β-Actin was used as a loading control. (**d**) qPCR analysis of Hif-1α mRNA levels in BMDMs transduced with lentiviral vectors expressing either a non-targeting control (shNTC) or two different shRNA sequences against mRNA of *Hif1a* gene (shHif-1α-1 and shHif-1α-2). Data are presented as mean±s.e.m of one representative of two independent experiments performed in technical triplicate. *** = P <0.001 (two-tailed Student's t-test) compared with shNTC BMDMs. (**e**) Effect of HIF-1α silencing on intracellular IL-1β mRNA levels in resting and LPS-activated BMDMs cultured for 24h in the presence or absence of 11 mM mannose in the medium. Data are presented as mean±s.e.m of one representative of two independent experiments performed in technical triplicate. *** = P <0.001 (two-tailed Student's t-test). Source data are provided as a Source Data file.

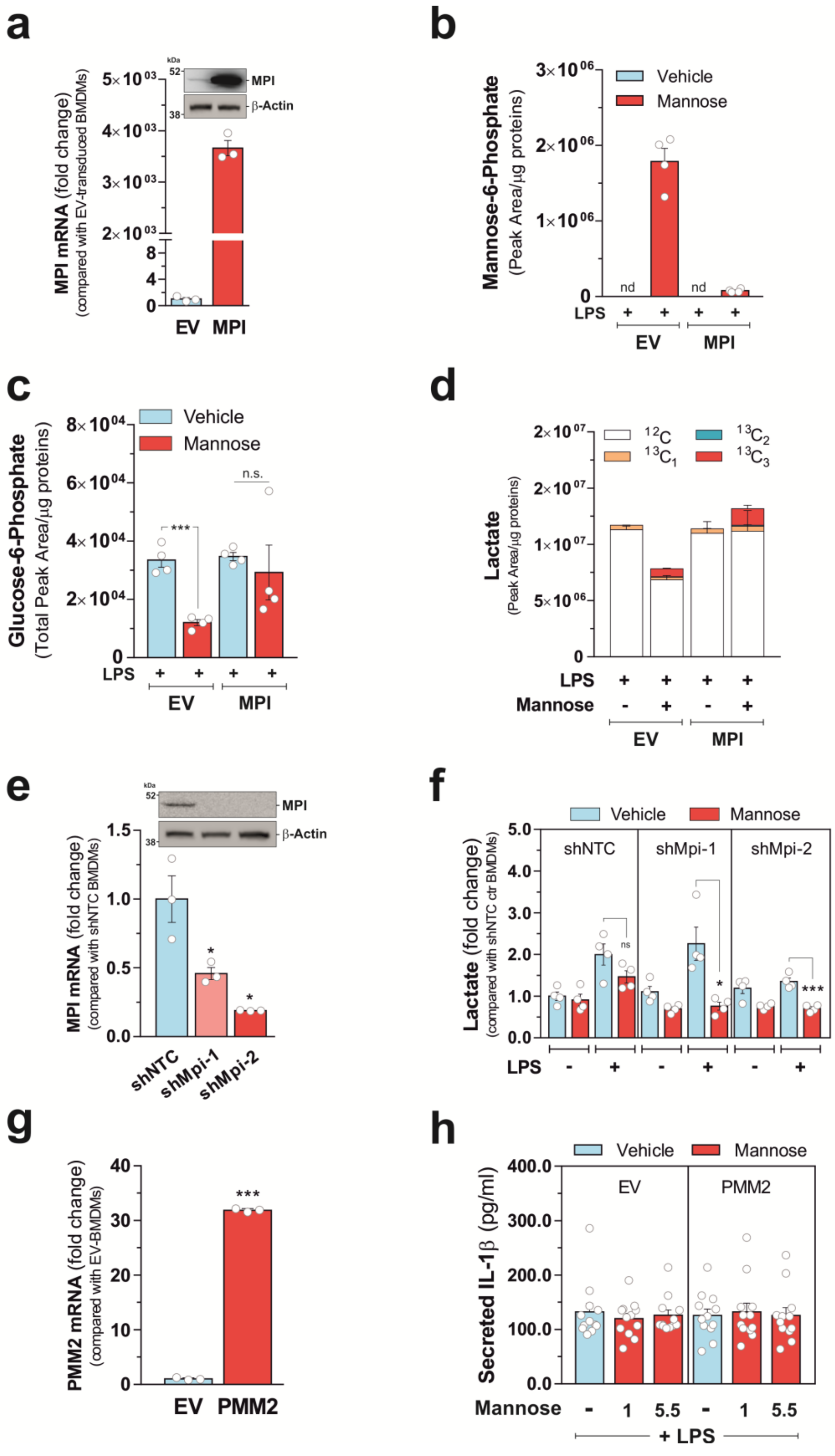

Supplementary Figure 6. MPI activity dictates response of LPS-activated macrophages to mannose. (**a**) qPCR analysis of MPI mRNA levels in BMDMs

transduced with an empty (EV) or a *Mpi* cDNA-overexpressing lentiviral vector (MPI). Top panel, representative image of MPI levels, measured by Western Blotting, in EV and MPI-transduced BMDMs. (**b-d**) Effects of MPI overexpression on the intracellular levels of the indicated metabolites in BMDMs stimulated with LPS for 24h in RPMI medium containing 11 mM glucose in the presence/absence of 11 mM U-$^{13}C_6$-Mannose. In (C) $^{13}C_6$-Mannose-6-Phosphate and ^{12}C-Glucose-6-Phosphate isolopologues were used as identifiers of the intracellular pools of Mannose-6-Phosphate and Glucose-6-Phosphate, respectively. (**e**) qPCR analysis of MPI mRNA levels in BMDMs transduced with lentiviral vectors expressing either a non-targeting control (shNTC) or two different shRNA sequences against mRNA of *Mpi* (shMpi-1 and shMpi-2). * = P <0.05 (two-tailed Student's t-test) compared to shNTC BMDMs. Top panel, representative image of MPI levels, measured by Western Blotting, in shNTC and MPI-silenced BMDMs. (**f**) Effect of MPI silencing in BMDMs transduced as in (e) on intracellular abundance of lactate measured in BMDMs cultured for 24h in RPMI medium containing 11 mM glucose in the presence/absence of 4 mM Mannose. (**g**) qPCR analysis of PMM2 mRNA levels in BMDMs transduced with an empty (EV) or a mouse *Pmm2* cDNA-overexpressing lentiviral vector (PMM2). (**h**) ELISA-mediated determination of IL-1β in culture media of BMDMs transduced as in (g) stimulated with LPS for 24h in RPMI medium containing 11 mM glucose in the presence/absence of the indicated concentrations (mM) of Mannose. In (a, e, g) β-Actin was used as a loading control. Data are presented as mean±s.e.m of: (a) one representative of two independent experiments performed in triplicate; (b-d) two independent experiments performed in duplicate, pooled together; (e) one representative experiment performed in triplicate; (f) two independent experiments performed in duplicate, pooled together; (g) Data are presented as mean±s.d. of one

representative of two independent experiments performed in triplicate; (h) n=12 wells pooled from two independent experiments. * = P <0.05; *** = P <0.001; (two-tailed Student’s t-test). Source data are provided as a Source Data file.

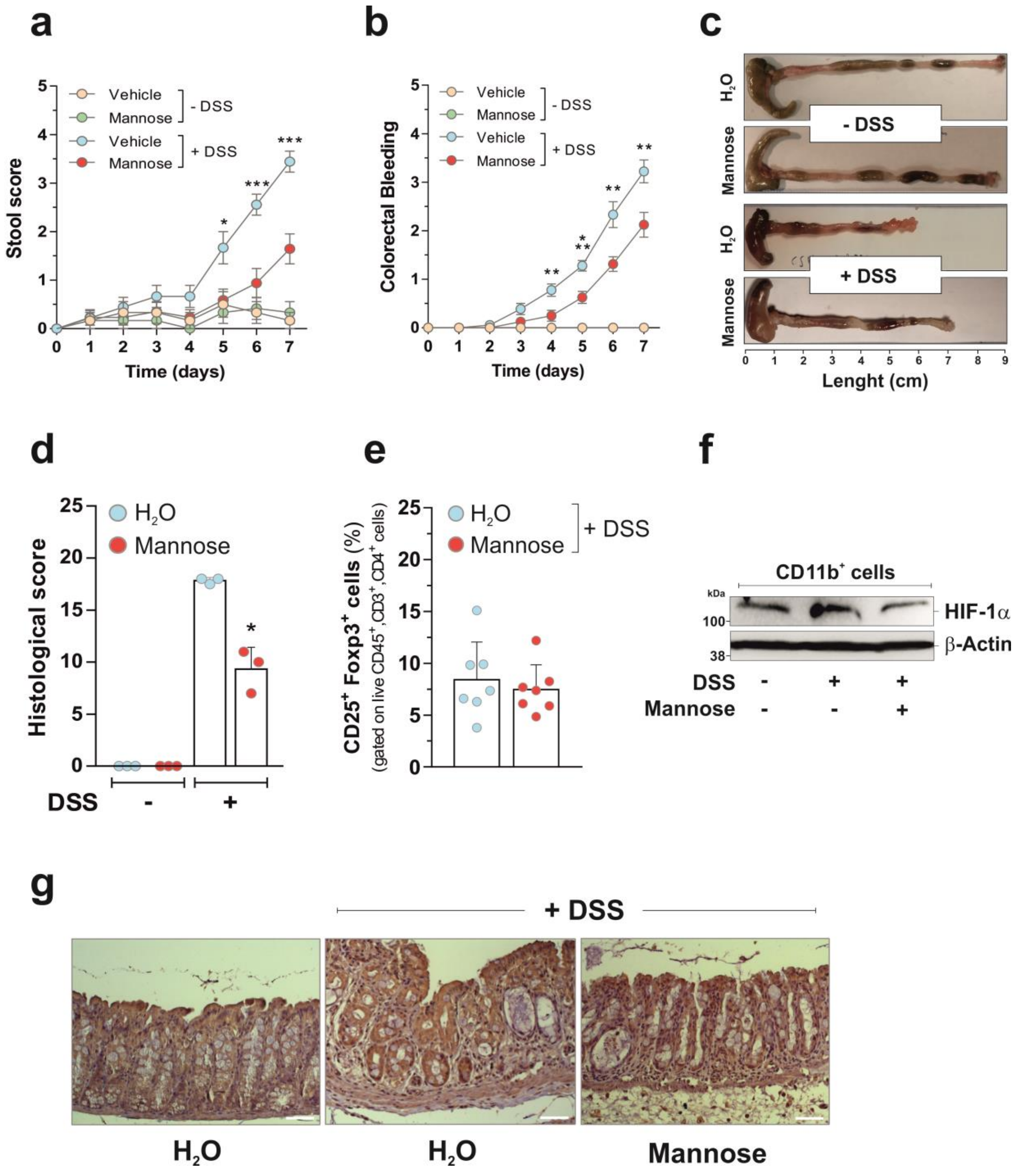

Supplementary Figure 7. Mannose protects mice from DSS-induced ulcerative colitis. Effect of mannose on stool (**a**) and colorectal bleeding (**b**) scores measured daily in mice described in Fig. 4A during DSS treatment schedule. Data are presented as mean±s.e.m of n=18 (DSS-treated) and n=12 (DSS-untreated) mice pooled from two independent experiments. * = P <0.05; ** = P <0.01; *** = P <0.001; (two-tailed Student's t-test) compared with mannose-untreated DSS-administered

mice. (**c**) Representative images of colons isolated from the indicated groups of mice described in Fig. 4A at the end of DSS administration. (**d**) Histological score for assessing the impact of mannose treatment on colitis extent in mice. Data are presented as mean±s.d. of n=3 mice per group. * = *P* <0.05 (two-tailed Student's t-test). (**e**) FACS-mediated analysis of T_{regs} infiltrating colon lamina propria of DSS-adminstered mice in response to mannose treatment, as described in (A), at the end of the experiment. Data are presented as mean±s.d. of n=7 mice per group. (**f**) Representative image of HIF-1□□levels of an experiment performed once, measured by Western Blotting, in CD11b$^+$ myeloid cells pooled from colons of the indicated group of mice (n=6 mice *per* group) treated as described in Fig. 4a. β-Actin was used as a loading control. (**g**) Representative figures out of three independently acquired showing the Immunohistochemical staining of HIF-1α in colon sections of the indicated groups of mice treated as described in Fig. 4a and collected at the end of the experiment (scale bars = 50 μm). Source data are provided as a Source Data file.

a

b

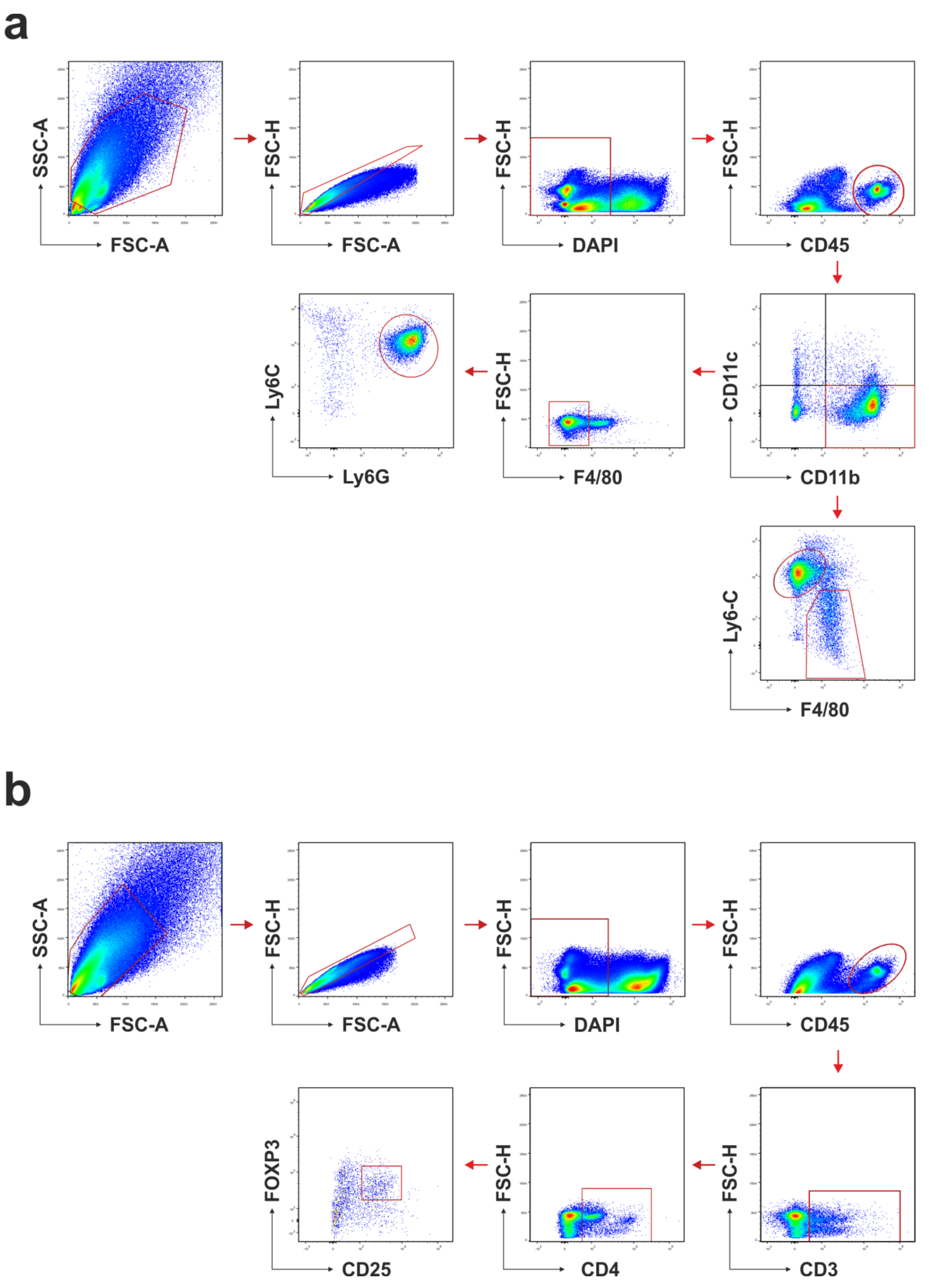

Supplementary Figure 8. Flow cytometry gating strategies. Representative gating strategies employed for FACS-mediated analysis of myeloid cell types (**a**) and T_{regs} (**b**) infiltrating colon lamina propria of DSS-adminstered mice in response to

mannose treatment, as described in Fig. 4e and Supplementary Fig. 7E, respectively. Cell populations were gated on dapi-negative (live) single cells and identified as follows: monocytes ($CD45^+$, $CD11b^+$, $CD11c^-$, $F4/80^-$, $Ly6C^+$), macrophages ($CD45^+$, $CD11b^+$, $CD11c^-$, $F4/80^+$, $Ly6C^-$), neutrophils ($CD45^+$, $CD11b^+$, $CD11c^-$, $F4/80^-$, $Ly6G^+$, $Ly6C^+$), regulatory T cells (T_{regs}, $CD45^+$, $CD3^+$, $CD4^+$, $CD25^+$, $Foxp3^+$).

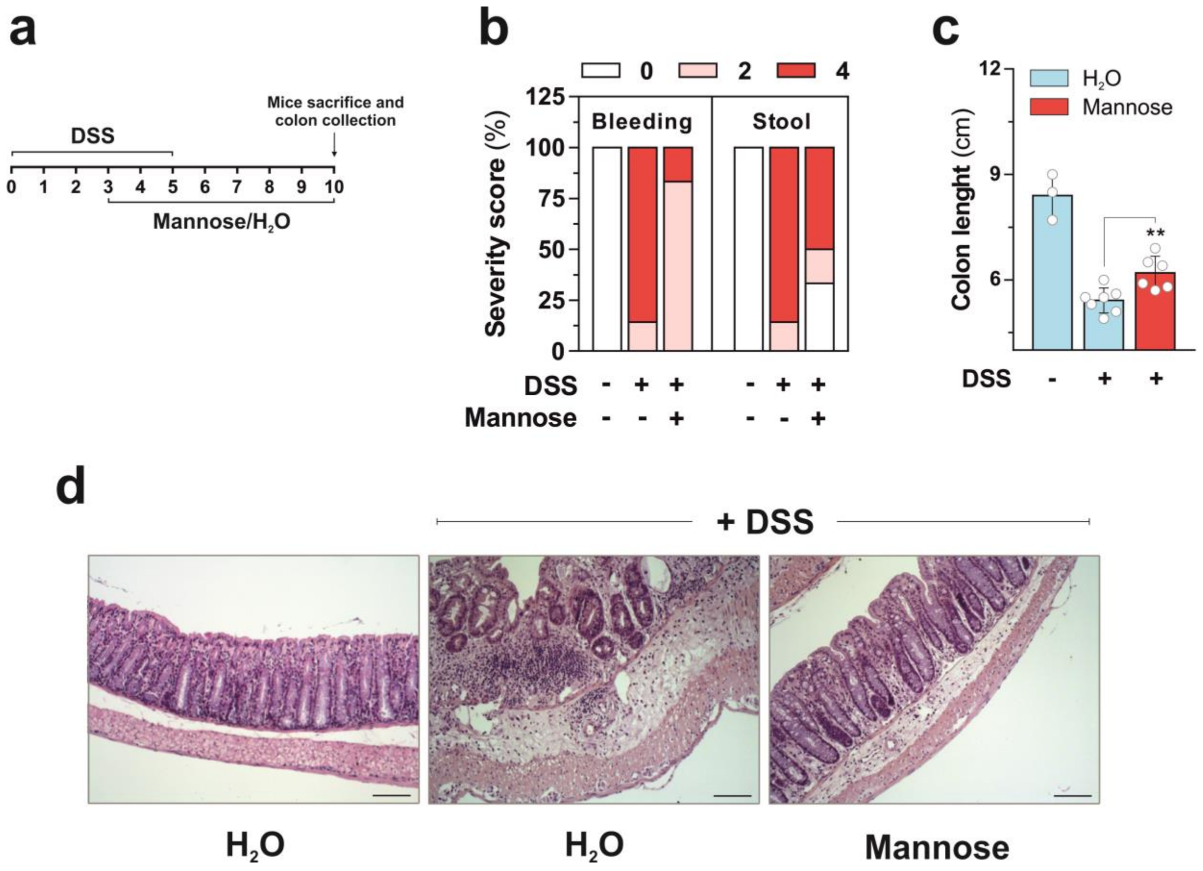

Supplementary Figure 9. Therapeutic efficacy of mannose against DSS-induced ulcerative colitis. (**a**) Seven-week-aged C57BL/6 mice were provided with 3% (w/v) DSS in drinking water *ad libitum* for 5 days and then returned to normal drinking water until the end of the experiment (day 10). Starting from day 3 of DSS administration - time sufficient to induce formation of soft stools and the appearance of occult blood in feces - mice were treated with 200 µl of either water (n=7 mice) or 20% (w/v) mannose (n=6 mice) by oral gavage twice a day, until the end of the experiment. (**b**) Fraction of mice, treated as described in (a), with the indicated colitis severity score. (**c**) Colon length of mice described in (a). Data are presented as mean±s.d. ** = P <0.01 (two-tailed Student's t-test). (**d**) Representative H&E-stained images out of three independently acquired of colon sections from the indicated groups of mice treated as in (a) at the end of the experiment (scale bar 100 µm.). Source data are provided as a Source Data file.

Gene Name	Application	Sequence
Il1b	qPCR	fw TGCCACCTTTTGACAGTGATG
Il1b	qPCR	rv ATGTGCTGCTGCGAGATTTG
Tnf	qPCR	fw GCCTCTTCTCATTCCTGCTT
Tnf	qPCR	rv TGGGAACTTCTCATCCCTTTG
Il6	qPCR	fw CGGCCTTCCCTACTTCACAA
Il6	qPCR	rv GCCATTGCACAACTCTTTTCTCA
Mpi	qPCR	fw ACCTTAGGCCAGTGGATTGC
Mpi	qPCR	rv TCTGCCAGCTCCTTGTTAGG
Actb	qPCR	fw CACACCCGCCACCAGTTCGC
Actb	qPCR	rv TTGCACATGCCGGAGCCGTT
Pmm2	qPCR	fw GATTATGTGTTTCCAGAGAATGGC
Pmm2	qPCR	rv CAGATGCCCTTGAATATTCTGCT
Mrc1	qPCR	fw GGCTGATTACGAGCAGTGGA
Mrc1	qPCR	rv CATCACTCCAGGTGAACCCC
IL1B	qPCR	fw AGAAGTACCTGAGCTCGCCA
IL1B	qPCR	rv CTGGAAGGAGCACTTCATCTGT
TNF	qPCR	fw CCCATGTTGTAGCAAACCCTC
TNF	qPCR	rv TATCTCTCAGCTCCACGCCA
Il10	qPCR	fw TGATGGGAGGGGTTCTTCCT
Il10	qPCR	rv AGGACACCATAGCAAAGGGC
Il1rn	qPCR	fw GCCCTTCTGGGAAAAGACCC
Il1rn	qPCR	rv GGCACCATGTCTATCTTTTCTTCT
RPS18	qPCR	fw CATTAAGGGTGTGGGCCGAA
RPS18	qPCR	rv TGGGATCTTGTACTGGCGTG
Mpi	Cloning	fw: GCGCCGACCGGTATGGCGAGTCCGCGAGT
Mpi	Cloning	rv: GGCGCCGTCGACCTACAGCAGACAGCAGGCCC
Pmm2	Cloning	fw: CGCCGACCGGTATGGCCACTCTCTGTCTCTTCGAC
Pmm2	Cloning	rv: GGCGCCGTCGACTCAAGGGAAGAGCCCCTCACAG

Supplementary Table. List of primers used in this study.
