# Supplementary material for: D-mannose suppresses macrophage IL-1β production
Source: Nat Commun. 2020 Dec 11;11:6343. doi: 10.1038/s41467-020-20164-6 (PMC7733482; doi:10.1038/s41467-020-20164-6)
Supplement: Supplementary file 2 — Reporting Summary [file 41467_2020_20164_MOESM2_ESM.pdf]

nature research

Corresponding author(s): Simone Cardaci

Last updated by author(s): Nov 2, 2020

Reporting Summary

Nature Research wishes to improve the reproducibility of the work that we publish. This form provides structure for consistency and transparency in reporting. For further information on Nature Research policies, see Authors & Referees and the Editorial Policy Checklist .

Statistics

For all statistical analyses, confirm that the following items are present in the figure legend, table legend, main text, or Methods section.

n/a	Confirmed	
☐	☒	The exact sample size (*n*) for each experimental group/condition, given as a discrete number and unit of measurement
☒	☐	A statement on whether measurements were taken from distinct samples or whether the same sample was measured repeatedly
☐	☒	The statistical test(s) used AND whether they are one- or two-sided<br>*Only common tests should be described solely by name; describe more complex techniques in the Methods section.*
☒	☐	A description of all covariates tested
☒	☐	A description of any assumptions or corrections, such as tests of normality and adjustment for multiple comparisons
☐	☒	A full description of the statistical parameters including central tendency (e.g. means) or other basic estimates (e.g. regression coefficient) AND variation (e.g. standard deviation) or associated estimates of uncertainty (e.g. confidence intervals)
☐	☒	For null hypothesis testing, the test statistic (e.g. *F*, *t*, *r*) with confidence intervals, effect sizes, degrees of freedom and *P* value noted<br>*Give P values as exact values whenever suitable.*
☒	☐	For Bayesian analysis, information on the choice of priors and Markov chain Monte Carlo settings
☒	☐	For hierarchical and complex designs, identification of the appropriate level for tests and full reporting of outcomes
☒	☐	Estimates of effect sizes (e.g. Cohen's *d*, Pearson's *r*), indicating how they were calculated

Our web collection on statistics for biologists contains articles on many of the points above.

Software and code

Policy information about availability of computer code

Data collection	FACSDIVA software (various versions) were used for flow cytometry data collection; 7500 Software 2 2.3 (Applied Biosystems) was used for qPCR data collection; Image Lab Touch Software 2.4 for Western Blotting data acquisition.
Data analysis	FlowJo v10 for flow cytometry data analysis; Thermo LCquan 2.7 (ThermoFisher Scientific) and MultiQuant 3.0.3 oftware packages were used for LC-MS data analysis; 7500 Software 2 2.3 (Applied Biosystems) was used for qPCR data analysis; Aperio ImageScope 12.1 (Leica Biosystem) was used for analysis of histological specimens; Image Lab Software 6.0.1. for Western Blotting data analysis; GraphPad Prism 7 and Microsoft Excel 2010 software packages were used for statistical analyses.

For manuscripts utilizing custom algorithms or software that are central to the research but not yet described in published literature, software must be made available to editors/reviewers. We strongly encourage code deposition in a community repository (e.g. GitHub). See the Nature Research guidelines for submitting code & software for further information.

Data

Policy information about availability of data

All manuscripts must include a data availability statement. This statement should provide the following information, where applicable:

- Accession codes, unique identifiers, or web links for publicly available datasets
- A list of figures that have associated raw data
- A description of any restrictions on data availability

The source data underlying Figs. 1a-d, 2a-g, 3a-f, 4a-c, e-h, and Supplementary Figs. 1a-j, 2a-b, 3a-c, 4a-f, 5a-e, 6a-h, 7a,b,d-f, 9b,c are provided as a Source Data file. All other data are available from the corresponding author upon reasonable requests.

Field-specific reporting

Please select the one below that is the best fit for your research. If you are not sure, read the appropriate sections before making your selection.

- [x] Life sciences
- [ ] Behavioural & social sciences
- [ ] Ecological, evolutionary & environmental sciences

For a reference copy of the document with all sections, see nature.com/documents/nr-reporting-summary-flat.pdf

Life sciences study design

All studies must disclose on these points even when the disclosure is negative.

Sample size	No statistical method was used to predetermine sample sizes.<br>For cell biology and metabolomics experiments, we chose sample sizes based on our previous experience (Cardaci et al., Nat Cell Biol, 2015; Gonzalez, P. S. et al. Nature, 2018). For experiments involving animal studies (in vivo and ex vivo samples collection, processing and analysis), sample sizes were determined on the basis of studies adopting similar experimental approaches to those described in our manuscript (Tannahill et al., Nature 2013; Mills et al., Cell 2016; Liu et al., Proc. Natl. Acad. Sci., 2016; Wirtz et al., Nature Protocols 2007; Singh et al., Immunity, 2014).
Data exclusions	No data were excluded from the analyses.
Replication	The number of times experiments were replicated in the laboratory is reported in each figure legend.
Randomization	Mice were randomly assigned to each experimental group.<br>For LC-MS data collection, sample runs were randomized in order to limit analytical variance due to retention time drifts.
Blinding	The investigators were blinded to allocation and outcome assessment of experiments involving animal studies. For experiments other than animal studies, blinding was not possible because data were largely analyzed by individual scientists who both collected and processed the samples.

Reporting for specific materials, systems and methods

We require information from authors about some types of materials, experimental systems and methods used in many studies. Here, indicate whether each material, system or method listed is relevant to your study. If you are not sure if a list item applies to your research, read the appropriate section before selecting a response.

Materials & experimental systems

n/a	Involved in the study	
☐	☒	Antibodies
☐	☒	Eukaryotic cell lines
☒	☐	Palaeontology
☐	☒	Animals and other organisms
☐	☒	Human research participants
☒	☐	Clinical data

Methods

n/a	Involved in the study	
☒	☐	ChIP-seq
☐	☒	Flow cytometry
☒	☐	MRI-based neuroimaging

Antibodies

Antibodies used

FITC-conjugated anti-mouse CD45 (clone 30-F11; Biolegend, 103108)
APC-conjugated anti-mouse CD11c (clone N418; Biolegend, 117309)
Pe-Cy7-conjugated anti-mouse/human CD11b clone (M1/70; Biolegend, 101215)
Ly6C PerCPCy5.5-conjugated anti-mouse Ly6C (clone HK1.4; Biolegend, 128011)
PE-conjugated anti-mouse F4/80 (clone BM8; Biolegend, 123109)
APC-Cy7-conjugated anti-mouse Ly6g (clone 1A8; Biolegend, 127623)
Brilliant violet 510-conjugated anti-mouse MHCII (clone M5/114.15.2; Biolegend, 107635)
APC-conjugated anti-mouse CD25 (clone PC61; Biolegend, 102011)
PerCPCy5.5-conjugated anti-mouse CD4 (GK1.5; Biolegend, 100433)
APC-Cy7-conjugated anti-mouse CD3e (clone 145-2C11; Biolegend, 100329)
Pe-Cy7-conjugated anti-mouse Foxp3 (clone FJK-16s, Ebioscience, 25-5773-80)
PE/Cyanine7-conjugated anti-mouse CD206 (clone C068C2, Biolegend, 141719)
Pacific Blue-conjugated anti-mouse/human CD11b (clone M1/70, Biolegend, 101223)
anti-HIF-1 alpha antibody (abcam ab2185)
anti-HIF-1 alpha (rabbit polyclonal, Novus Biologicals, NB100-499),

	anti-HIF-1 alpha (mouse monoclonal, BD Biosciences, clone 54/HIF-1α, 610958)<br>anti-MPI (mouse monoclonal, Santa Cruz Biotechnology, sc-393477)<br>anti-Vinculin (mouse monoclonal, Sigma-Aldrich, clone VIN-11-5, SAB4200729)<br>anti-β-Actin (mouse monoclonal, Sigma-Aldrich, clone AC-15, A5441)
Validation	All antibodies employed in our manuscript were previously reported and routinely used for the application herein used.<br>Antibodies employed for flow cytometry studies have been validated using several types of positive and negative controls. Positive controls included cell suspensions deriving from mouse bone marrow and spleen isolation/dissociation and purified macrophages. Negative controls included fluorescence minus one controls (FMO) and/or unstained samples.<br>All IHC antibodies were titrated on positive control tissue, starting from the recommended dilution. Respective staining without primary antibodies were used as a negative control.<br>All vendors used (Biolegend, eBiosciences, Abcam, Novus Biologicals, BD Biosciences, Santa Cruz Biotechnology and Sigma-Aldrich) report taking quality control measures to ensure that all antibodies sold are valid and reproducible. Validation information and citations for each antibody used herein could be found in the corresponding data sheets provided by the commercial supplier and visiting the following on-line repositories: https://www.biocompare.com/Antibodies/; https://www.antibodypedia.com/).

Eukaryotic cell lines

Policy information about cell lines

Cell line source(s)	All cell lines (HEK293T, SKOV3, RKO, SAOS2, U2OS, RAW 264.7) cells were originally obtained from the American Type Culture Collection (ATCC) repository.
Authentication	None of the cell line used were authenticated
Mycoplasma contamination	Cells were tested negative for Mycoplasma contamination
Commonly misidentified lines<br>(See ICLAC register)	No commonly misidentified cell lines were used in the study

Animals and other organisms

Policy information about studies involving animals; ARRIVE guidelines recommended for reporting animal research

Laboratory animals	7-9-week-old C57BL/6N (female and male) and Balb/c (male) mice were used.
Wild animals	The study did not involve wild animals
Field-collected samples	The study did not involve samples collected from the field
Ethics oversight	All experimental animal procedures were approved by the Institutional Animal Committee of the San Raffaele Scientific Institute.

Note that full information on the approval of the study protocol must also be provided in the manuscript.

Human research participants

Policy information about studies involving human research participants

Population characteristics	Healthy european volunteers (male and female, age 25-50 years).
Recruitment	Volunteers were recruited via the San Raffaele Hospital
Ethics oversight	Human peripheral blood was collected upon informed consent from healthy volunteers according to the Institutional Ethical Committee approved protocol (TIGET09) and with the Declaration of Helsinki.

Note that full information on the approval of the study protocol must also be provided in the manuscript.

Flow Cytometry

Plots

Confirm that:

- [x] The axis labels state the marker and fluorochrome used (e.g. CD4-FITC).
- [x] The axis scales are clearly visible. Include numbers along axes only for bottom left plot of group (a 'group' is an analysis of identical markers).
- [x] All plots are contour plots with outliers or pseudocolor plots.
- [x] A numerical value for number of cells or percentage (with statistics) is provided.

Methodology

Sample preparation	Biological sources, tissue processing steps and preparation of samples used for flow cytometry analyses were described in the Methods section of the manuscript (pages 19 -31)
Instrument	All samples were acquired using BD FACSCanto II.
Software	All samples were analysed by Flowjo v10 software.
Cell population abundance	Purity of Immuno-magnetically sorted and in vitro differentiated cells populations was confirmed by flow cytometry
Gating strategy	Immune cell populations isolated from mouse colons were gated on live (DAPI-) single cells and identified as follow:<br>monocytes (CD45+, CD11b+, CD11c-, F4/80-, Ly6C+),<br>macrophages (CD45+, CD11b+, CD11c-, F4/80+, Ly6C-)<br>neutrophils (CD45+, CD11b+, CD11c-, F4/80-, Ly6G+, Ly6C+).<br>Tregs (CD45+, CD3+, CD4+, CD25+, Foxp3+)<br>Fuorescence minus one controls (FMO) and/or unstained samples were used for gating. Gating strategies are shown in Supplementary file 8.

[x] Tick this box to confirm that a figure exemplifying the gating strategy is provided in the Supplementary Information.
